# *SEPALLATA1/2*-suppressed mature apples have low ethylene, high auxin and reduced transcription of ripening-related genes

**DOI:** 10.1093/aobpla/pls047

**Published:** 2012-12-13

**Authors:** Robert J. Schaffer, Hilary S. Ireland, John J. Ross, Toby J. Ling, Karine M. David

**Affiliations:** 1 The New Zealand Institute of Plant and Food Research, Private Bag 92169, Auckland 1142, New Zealand; 2 School of Biological Sciences, University of Auckland, Private Bag 92019, Auckland 1142, New Zealand; 3 School of Plant Science, University of Tasmania, GPO Box 252-55, Hobart, Tasmania 7001, Australia

## Abstract

**Background and aims:**

Fruit ripening is an important developmental trait in fleshy fruits, making the fruit palatable for seed dispersers. In some fruit species, there is a strong association between auxin concentrations and fruit ripening. We investigated the relationship between auxin concentrations and the onset of ethylene-related ripening in *Malus* × *domestica* (apples) at both the hormone and transcriptome levels.

**Methodology:**

Transgenic apples suppressed for the *SEPALLATA1/2* (*SEP1/2*) class of gene (*MADS8/9*) that showed severely reduced ripening were compared with untransformed control apples. In each apple type, free indole-3-acetic acid (IAA) concentrations were measured during early ripening. The changes observed in auxin were assessed in light of global changes in gene expression.

**Principal results:**

It was found that mature *MADS8/9-*suppressed apples had a higher concentration of free IAA. This was associated with increased expression of the auxin biosynthetic genes in the indole-3-acetamide pathway. Additionally, in the *MADS8/9*-suppressed apples, there was less expression of the *GH3* auxin-conjugating enzymes. A number of genes involved in the auxin-regulated transcription (*AUX/IAA* and *ARF* classes of genes) were also observed to change in expression, suggesting a mechanism for signal transduction at the start of ripening.

**Conclusions:**

The delay in ripening observed in *MADS8/9*-suppressed apples may be partly due to high auxin concentrations. We propose that, to achieve low auxin associated with fruit maturation, the auxin homeostasis is controlled in a two-pronged manner: (i) by the reduction in biosynthesis and (ii) by an increase in auxin conjugation. This is associated with the change in expression of auxin-signalling genes and the up-regulation of ripening-related genes.

## Abstract (precis)

Free auxin and expression of auxin-related genes were measured at ripening in *MADS8* suppressed apple fruit. It was found that the delayed ripening in these fruit was associated with high auxin and changes in auxin homeostasis and response genes.

## Introduction

**Fig. 1:**
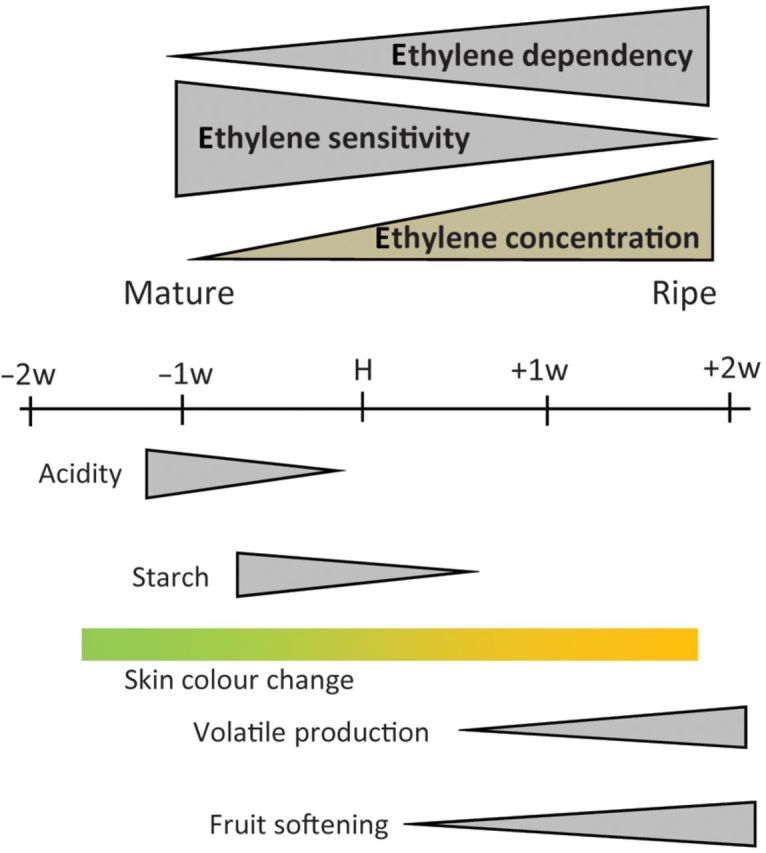
**Apple fruit ripening working model.** Mature apple fruit progress through a series of physiological changes, with differences in their sensitivity to and dependency on ethylene.

Fruit ripening is associated with dramatic changes in fruit physiology. In *Malus* × *domestica* (apple), ripening is associated with a respiratory climacteric and an increase in ethylene. This coincides with a conversion of starches to sugars, a reduction in fruit acids, a change in background skin colour from green to yellow, a softening of the flesh and an increase in flavour and aroma volatiles. If the effect of ethylene is removed, either by the use of 1-methylcyclopropene or through the suppression of genes associated with ethylene biosynthesis, many of these ripening changes are suppressed (Dandekar *et al.* 2004; Schaffer *et al.* 2007; Johnston *et al.* 2009). Using transgenic apple lines suppressed for a gene involved in ethylene biosynthesis, *ACC OXIDASE 1* (*ACO1*), a model was proposed whereby increasing the concentrations of ripening-associated ethylene (type II) controls a sequential progression of events starting with starch/acid changes, followed by skin colour changes and culminating in flesh softening and aroma volatile production (Johnston *et al.* 2009) (Fig. 1).

While many ripening characters are dependent on ethylene, other developmental factors within the fruit can control early ripening events and it has been shown that *MADS*-box transcription factors are playing an important role in this process. The *SEP4*-like *RIPENING INHIBITOR* (*RIN*) gene in tomato (Vrebalov *et al.* 2002) and the *SEP1/2*-like gene, *FaMADS9*, in strawberry have been shown to play a key role in the developmental control of fruit ripening (Seymour *et al.* 2011) as down-regulation of these genes inhibits all ripening-related traits. In apple, we have recently shown that suppression of the *SEP1/2*-like genes *MADS8/9* can also inhibit all ripening events (Ireland *et al.* 2013).

There is now a growing body of literature demonstrating the complex interaction of plant hormones with one another (Vandenbussche and Van Der Straeten 2007; Kuppusamy *et al.* 2009; Ross *et al.* 2011), which suggests that individual hormones do not act independently. The interactions between ethylene and auxin are complex and depend on both developmental stage and cell/tissue type; for example, they are each able to induce the other's biosynthesis only in certain temporal and spatial contexts (Trainotti *et al.* 2007; Vandenbussche *et al.* 2012). It has previously been shown that in fruits which have ripening traits that are less dependent on ethylene (often referred to as non-climacteric fruits), auxin plays a role in inhibiting the fruit ripening effect. In strawberries, for example, removal of the seed from developing fruit can cause early ripening, which can be repressed by the addition of exogenous auxins (Given *et al.* 1988). The reduction of auxin concentrations before the initiation of ripening has been observed in a number of fruits, and has been suggested to be a prerequisite for ripening to occur (Given *et al.* 1988; Buta and Spaulding 1994; Purgatto *et al.* 2002; Zaharah *et al.* 2012). It has been proposed that this reduction in auxin concentration is controlled by the action of the GH3 class (group II) of auxin conjugation enzymes. Indeed, other non-climacteric fruits such as grapes (Bottcher *et al.* 2010) and capsicum (Liu *et al.* 2005) have high levels of GH3 expression at fruit maturity. Surprisingly, when capsicum *GH3* is over-expressed in tomato, an ethylene-requiring fruit (climacteric), the fruit are able to ripen more rapidly when exogenous ethylene is applied (Liu *et al.* 2005). This demonstrates the complexity of auxin action, where different concentrations can lead to different physiological responses (Ross *et al.* 2011). In this case it points to low auxin-facilitating ethylene action. The expression patterns of the *GH3* class of gene in apple are consistent with a low auxin requirement for ripening, where there is an increase in two *GH3*-like genes (*GH3.1* and *GH3.101*) at fruit maturation (Devoghalaere *et al.* 2012).

While fruit ripening in many species has been shown to be controlled through ethylene, auxin and developmental pathways, the links between these pathways have not yet been established. In this study, the role of auxin in *MADS8/9*-suppressed apple fruit (*MADS8/9as*) was assessed by comparing free indole-3-acetic-acid (IAA) concentrations and the associated changes in gene expression at fruit maturity.

## Methods

### Apple tree growth conditions

All apple plants were grown in contained greenhouse conditions, with *Malus* × *domestica* ‘Royal Gala’ controls grown next to *M. domestica* ‘Royal Gala’ transformed with an antisense construct for the *MADS8* gene. In one line *MADS8as-9* both *MADS8* and *MADS9* homeologues were suppressed (Ireland *et al.* 2013) and it was subsequently named *MADS8/9as*. Flowers of both the ‘Royal Gala’ control and *MADS8/9as* were pollinated at ‘full bloom’ with pollen from ‘Granny Smith’. When the ‘Royal Gala’ control fruit began to show a change in background skin colour, and representative fruit had a starch index of 1–2 (0–7 scale), all fruit were harvested for both genotypes.

### Auxin concentration assessments

Tissues from each part of the apple (skin, cortex and core) were collected at fruit maturity and 2 weeks after harvest. All samples were frozen immediately in liquid nitrogen and stored at −80 °C. Free IAA concentration was assessed using methods described in Devoghalaere *et al*. (2012).

### RNA extraction and mRNA-seq analysis

RNA was extracted from mature apples left to ripen for 4 days at 20 °C. Individual tissues from apple fruit (skin, cortex and core) were collected in liquid nitrogen and stored at −80 °C until needed. RNA extraction was conducted using the method described in Schaffer *et al*. (2007). RNA was cleaned using Qiagen cleanup columns as per the manufacturer's instructions. RNA from each tissue type was combined in equal quantities to produce a pool of ‘Royal Gala’ RNA and *MADS8/9as* RNA. Ten micrograms of the pooled RNA were sequenced at the Australian Genomics Resource Facility (Brisbane, Australia) using Illumina HiSeq 2000. These samples were two of a total of nine barcoded samples used in a single lane. A total of 7.8 and 8.1 M, 100-bp reads were obtained from the ‘Royal Gala’ pooled fruit tissue and the *MADS8as* pooled fruit tissue, respectively.

The frequency of gene expression was assessed using the ShortRead package (Morgan *et al.* 2009) in a bioconductor (Gentleman *et al.* 2004) using the statistical software package ‘R’. A Pdict library of each of the sequencing reads was created using the 5–40 bp region of each of the 100-bp reads. Any Pdict library containing the ambiguous nucleotide ‘N’ in this region was removed before analysis. A sequence frequency was obtained for each *Malus* × *domestica* gene model (Velasco *et al.* 2010) for each Pdict library. Frequencies were normalized by dividing the number of reads by the total number of reads in each cleaned Pdict library and dividing by the length of the predicted gene, giving a value of RPMK (reads per million per kb). Four of these key genes were confirmed using qPCR [see Additional Information 1].

## Results

### Auxin concentration during fruit maturation

**Fig. 2:**
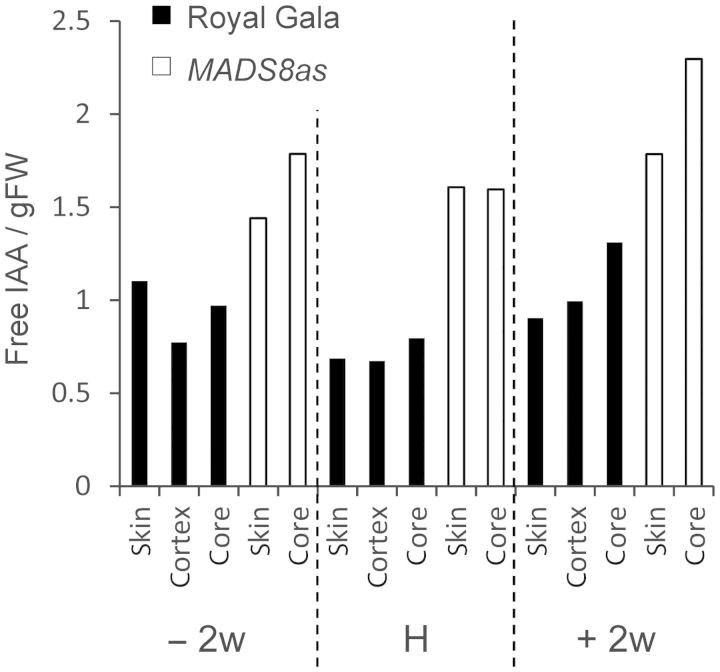
**Free auxin content in mature *MADS8/9as* and ‘Royal Gala’ apples.** Changes in free IAA content (ng g^−1^ fresh weight; FW) in different tissue zones of the fruit (core, cortex, skin), 2 weeks before full maturity (−2w), at maturation (H: harvest) and at an early stage of ripening (+2w: +2 weeks). Measurements were performed on a pool sample of 10 fruit.

Suppression of *MADS8/9* causes delayed ripening; one line *MADS8/9as* is particularly severe. To assess the concentrations of auxin in mature to ripe apples, ‘Royal Gala’ control and *MADS8/9as* apples were harvested 2 weeks before the ‘Royal Gala’ apples reached maturity, at maturity and 2 weeks after harvest. Free IAA in *MADS8/9as* apples was considerably higher, and had a more constant level than those observed in ‘Royal Gala’ at all time points (Fig. 2). In ‘Royal Gala’ tissue, IAA dropped towards harvest and then increased as ripening progressed. This drop in IAA was not observed in the *MADS8/9as* fruit.

### Transcriptome analysis of auxin-related genes

**Fig. 3:**
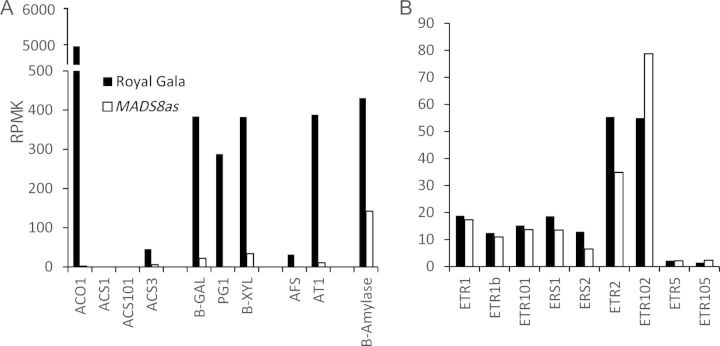
**Expression of ethylene-related genes.** Expression of ethylene-related genes in early ripening ‘Royal Gala’ control apples and *MADS8/9as* apples harvested at the same day post pollination and kept for 4 days after harvest. RPMK, reads per million per kb.

Transcriptional changes occur before phenotypic changes; therefore an early ripening time point (4 days following harvest) was chosen for transcriptomic analysis. Using the predicted gene models from the apple genome (Velasco *et al.* 2010), expression levels were calculated based on frequency of sequence reads [see Additional Information 2]. Expression levels of key ripening genes were compared. These included the ethylene biosynthesis gene *ACC OXIDASE1* (*ACO1*), *ACC SYNTHASE 1* (*ACS1*), cell wall hydrolyases *POLYGALACTURONASE1* (*PG1*), *β-GALACTOSIDASE1* (*β-GAL1*), and flavour biosynthesis genes α-*FARNESENE SYNTHASE1* (*AFS*), *ACYLTRANSFERASE1* (*AT1*). The levels of expression of all these genes were consistent with an ‘early ripening’ stage of development in ‘Royal Gala’ (Fig. 3A). All of these key genes had a higher expression in the ‘Royal Gala’ control compared to the *MADS8/9as* fruit.

It has been postulated that, in plants, production of 1-aminocyclopropane-1-carboxylic acid (ACC) by ACC SYNTHASE (ACS) is the rate-limiting step in ethylene biosynthesis (Argueso *et al.* 2007). In ‘Royal Gala’, fruit ripening is associated with an initial increase in *ACS3*, followed by a sustained increase in *ACS1* (Wang *et al*. 2009). In the *MADS8/9as* apples, there was a much lower level of *ACS3* than in ‘Royal Gala’ (Fig. 3A), and no detectable transcripts of *ACS1* compared with low expression in ‘Royal Gala’. The ethylene receptors *ETHYLENE RESPONSE SENSOR 1 (ERS1)* and *ETHYLENE RESPONSE 2* (*ETR2*), which have been shown to be induced by ethylene (Ireland *et al.* 2012), had low expression in both fruit at this time point (Fig. 3B).

### Expression of auxin homeostasis genes from apple

**Fig. 4:**
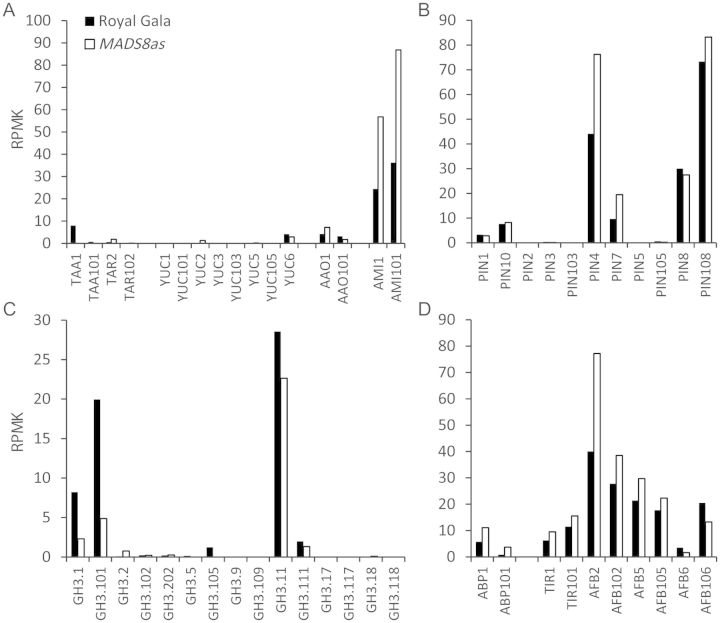
**Expression of genes involved in auxin homeostasis and auxin perception.** Expression of auxin-related genes in early ripening ‘Royal Gala’ control apples and *MADS8/9as* apples harvested at the same day post pollination and kept for 4 days after harvest.

While the ethylene biosynthesis and response pathway in ripening apples has been well characterized, the auxin biosynthetic genes in apple fruit have not. Two pathways have been proposed for the production of IAA: a tryptophan (Trp)-dependent pathway and a Trp-independent pathway (Normanly *et al.* 1993). The genes involved in the Trp-independent pathway have not been described, while the Trp-dependent pathway is better understood. The production of IAA from Trp can potentially take three routes in plants: (i) the CYP79B2/B3 pathway (CRYTOCHROME P450 79B2/79B3), which is specific to Brassicaceae (Zhao *et al.* 2002); (ii) the TAA1-YUC pathway (TRYPTOPHAN AMINOTRANSFERASE OF ARABIDOPSIS 1/YUCCA), in which TAA1 (or TAR, TAA-RELATED proteins) is responsible for the production of indole-3-pyruvic acid (IPyA) and YUCs function downstream in a series of reactions to convert IPyA into IAA (Stepanova *et al.* 2011); (iii) the indole-3-acetamide (IAM) pathway, for which the enzyme responsible for the conversion from Trp to IAM is not known but conversion from IAM to IAA involves the enzyme INDOLE-3-ACETAMIDE HYDROLASE (AMI) (Nemoto *et al.* 2009). Genes were mined from the TAA1-YUC and IAM pathways by comparing the apple gene models to the closest *Arabidopsis thaliana* (Arabidopsis) homologues. As the apple genome results from an ancient duplication, it is common to find two homologous genes that align closely in a phylogenetic tree. When homologous genes were identified, we followed the naming convention used by Devoghalaere *et al*. (2012). In apple there are four putative *TAA1/TAR* genes and eight putative *YUC* genes from the TAA1-YUC pathway (Table 1), all of which have low expression in mature apple fruit (Fig. 4A). In the IAM pathway, we identified two putative homologues for *AMI*: *AMI1* and *AMI101* (Table 1), which are both expressed highly in mature apples (Fig. 4B), suggesting that this is the predominant pathway for IAA in mature apple fruit. Expression of the AMI genes is higher in the *MADS8/9as* apples than in the control, possibly explaining the high concentrations of IAA in the *MADS8/9as* fruit.

**Table 1:** Auxin synthesis genes.

Gene name	Linkage group	Accession
*TAA1*	16	MDP0000616079
*TAA101*	13	MDP0000310220
*TAR2*	6	MDP0000267098
*TAR102*	14	MDP0000203835
*YUC1*	12	MDP0000228434
*YUC101*	15	MDP0000126406
*YUC 2*	2	MDP0000719430
*YUC 3*	13	MDP0000437311
*YUC 103*	17	MDP0000471028
*YUC 5*	5	MDP0000727457
*YUC 105*	10	MDP0000155609
*YUC 6*	15	MDP0000468914
*AMI1*	4	MDP0000295299
*AMI101*	12	MDP0000872599

Other auxin homeostasis genes previously mined from the apple genome (Devoghalaere *et al.* 2012) were screened for differences in expression (Fig. 4B). These included the transport genes (*PIN*) and conjugation enzymes (*GH3*). From these gene families, there was a higher level of *PIN4* and *PIN7* expression in the *MADS8/9as* fruit and higher amounts of *GH3.1* and *GH3.101*-conjugating enzymes in the ‘Royal Gala’ control.

### Expression of auxin-signalling genes

**Fig. 5:**
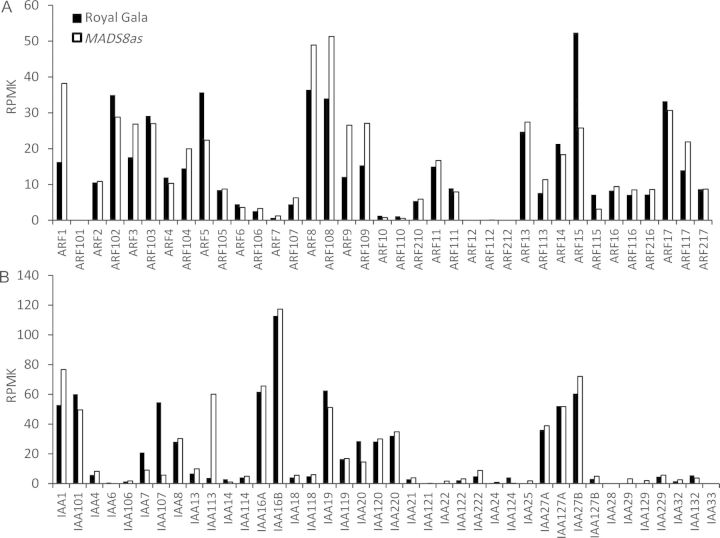
**Expression of auxin-signalling genes.** Expression of auxin-signalling genes in early ripening ‘Royal Gala’ control apples and *MADS8/9as* apples harvested at the same day post pollination and kept for 4 days after harvest.

Auxin-related signalling genes previously mined from the apple genome (Devoghalaere *et al.* 2012) were screened for differences in expression (Figs 4D and 5). While many of these auxin-signalling genes showed very similar expression patterns between the *MADS8/9as* apples and ‘Royal Gala’ controls, a few showed high expression levels (>10 RPMK) and a significant change in expression. All the perception genes appeared to be up-regulated in the *MADS8/9as* apples, with *AUXIN BINDING PROTEIN 1* (*ABP1)* and *AUXIN SIGNALING F-BOX 2 (AFB2)* having a 1.9-fold increase in expression (Fig. 4D). At the transcriptional regulation level for the *ARF/IAA* class of genes, *IAA113* showed a 15.8-fold up-regulation in expression in the *MADS8/9as* apples and *IAA107* showed a 9.6-fold up-regulation in ‘Royal Gala’ fruit (Fig. 5). *ARF1* and *ARF9* showed 2.3- and 2.1-fold higher expression in the *MADS8/9as* fruit, respectively, while *ARF15* showed a 2-fold increase in ‘Royal Gala’.

### Global changes in expression patterns of other genes

The severe ripening inhibition phenotype in *MADS8/9as* fruit compared with ‘Royal Gala’ was also observed at the transcriptional level, with many ripening-related genes such as the cell wall genes *PG1* and *β-GAL1*, and aroma volatile genes *AFS* strongly up-regulated in the ‘Royal Gala’ controls compared with the *MADS8/9as* fruit (Fig. 3). To identify other genes that were potentially involved in ripening, the expression levels of genes from the two lines were compared.

Of the 95 216 gene models for apple, 2650 were more than 2-fold up-regulated in the ‘Royal Gala’ apples, and 5675 more than 2-fold up-regulated in *MADS8/9as* apples [see Additional Information 2]. When the most extreme changes were considered, there was a >10-fold change in expression. In the ‘Royal Gala’ apples, 146 genes were up-regulated to this level; many have been previously published as ripening related [see Additional Information 3]. When grouped into general classes based on homology, the largest group would be genes involved in secondary metabolism, such as the aroma volatile genes *ALCOHOL DEHYDROGENASE* (*ADH*) and *LIPOXYGENASE* (*LOX*), as well as the cell-wall-related genes (some already described in the previous section; Table 2). A total of 105 genes up-regulated in the *MADS8/9as* fruit included a large class of photosynthetic genes (Table 2). It is likely that rather than being up-regulated, these genes are down-regulated in the early ripening ‘Royal Gala’ apples. Consistent with the high concentrations of IAA, also within the up-regulated genes in the *MADS8/9as* apples is a *GIBBERELLIN 20 OXIDASE* biosynthesis enzyme (Lemaire-Chamley *et al.* 2005). In addition, dehydration genes are differentially expressed. This is not surprising, as the apples were sampled following 4 days at room temperature. What is of interest is that two different sets of dehydration-related genes were affected in the *MADS8/9as* apples compared with the ‘Royal Gala’ controls, suggesting that the two fruit lines have distinct pathways of coping with water loss following harvesting.

**Table 2:** Summary of mRNA-seq gene classes.

	>10-fold induced ‘Royal Gala’	>10-fold induced *MADS8as-9*
Cell wall	11	0
Secondary metabolism	64	13
Allergen	6	0
Regulation	5	7
Hormone	8	2
Auxin	8	1
Gibberellins	0	1
Transcription factor	4	6
Dehydration	5	6
Photosynthesis	0	16
Others	43	55

## Discussion

In many fruit species, ripening is controlled through ethylene and/or auxin concentrations. Recent studies are beginning to establish a link between these two hormones through the conjugation of free IAA by the GH3 family of enzymes (Liu *et al.* 2005). The lack of ripening observed in the *MADS8/9as* apples has previously been linked to a low ethylene production, with concentrations of ethylene barely detectable in the *MADS8/9as* fruit, similar to the *ACO1* knockout apples (Ireland *et al.* accepted). Here we present data that may suggest a second way in which ripening is inhibited in these fruit, through the maintenance of high auxin concentrations. The suppression of the *MADS8/9* genes results in high auxin concentration, at amounts similar to those observed in rapidly expanding apple fruit (Devoghalaere *et al.* 2012). Molecular analysis presented here suggests that this is achieved by a combination of a high level of expression of auxin synthesis genes and a lack of expression of the auxin-conjugating enzymes *GH3.1* and *GH3.101*.

There is now a growing body of literature showing that hormones do not act independently of one another, with strong interactions between auxin and ethylene, and auxin and gibberellins (Ross *et al.* 2011). The auxin relationship with ethylene is complex and, so far, models of interaction have been established during vegetative growth (Ross *et al.* 2011; Vandenbussche *et al.* 2012). Low auxin concentrations seem to be required at the onset of ripening to promote ethylene action; this might seem counterintuitive, as auxin has also long been known to promote ethylene biosynthesis (Nakagawa *et al.* 1991; Liang *et al.* 1992). However, an analogy can be made to the leaf abscission process, where auxin and ethylene have opposite effects, i.e. ethylene will promote abscission, while auxin will delay it (Bleecker and Patterson 1997). The generally accepted model is that a basipetal IAA flux through the abscission zone, i.e. maintaining high IAA concentrations, prevents abscission by rendering the abscission zone insensitive to ethylene (Roberts *et al.* 2002).

The synthesis of auxin in Arabidopsis has been mapped to three pathways, one that appears to be specific to *Brassica* species. There are few studies in the literature that examine the pathways that regulate auxin concentration in fruits. In this study, based on expression patterns it appears that, in the Trp-dependent pathway, auxin synthesis in apple fruit is through the IAM pathway. This is consistent with a microarray experiment performed on developing tomato fruit that showed that a tomato *AMI*-like gene is up-regulated in expanding tomato locular tissue (Lemaire-Chamley *et al.* 2005), and this pathway is the dominant pathway in developing pea pods (Tivendale *et al.* 2012). An earlier report looking specifically at AMI activity in different tissues of trifoliate orange led to the conclusion that the IAM pathway for auxin biosynthesis was occurring in the very young fruit of *Poncirus trifoliata* but only transiently (Kawaguchi *et al.* 1993).

This study suggests that in apple the *MADS* box, *SEP* class of genes can regulate the concentrations of IAA, either directly or indirectly. In untransformed ‘Royal Gala’, the expression pattern of *MADS8/9* shows high expression early in fruit development, which then decreases as the fruit expands. The expression then increases as the fruit matures (Ireland *et al.* accepted). Auxin concentrations in ‘Royal Gala’ apples show an inverse correlation to this expression pattern, peaking during the cell expansion phase (Devoghalaere *et al.* 2012). Interestingly, down-regulation of the homologue of *MADS8/9* in tomato, *TM29*, led to the production of parthenocarpic fruit (Ampomah-Dwamena *et al.* 2002). While IAA content had not been measured in the fruit, one possibility would be that parthenocarpy might result from high auxin concentration. While this is a new observation for *SEP* gene function, other *MADS* box genes have been shown to affect auxin local concentration indirectly by affecting the auxin transporter, PIN1, during silique maturation (Sorefan *et al.* 2009). The data showed that a local low auxin concentration is required for dehiscence of the Arabidopsis silique valves at pod maturity (Sorefan *et al.* 2009).

## Conclusions and forward look

This study demonstrates a strong link between the *MADS8/9* gene and a low auxin concentration through transcriptional control of biosynthesis and conjugating enzymes at fruit maturation. This will be an additional ripening inhibition signal and ties in with the lack of ripening phenotype in these apples. Future work is needed in apples to establish how ethylene interacts with auxin during the ripening process.

## Additional information

The following additional information is available in the online version of this article –

**File 1.** qPCR confirmation of mRNA-seq results of four key genes mentioned in this study.

**File 2.** mRNA-seq-based expression of the different apple gene models in mature ‘Royal Gala’ apples and *MADS8/9as* harvested at the same time post pollination.

**File 3.** Table of genes showing a 10-fold change in gene expression between ‘Royal Gala’ control and *MADS8/9as* apples.

## Sources of funding

This work was funded by the New Zealand Ministry of Science and Innovation FRST contract C06X0705; Pipfruit, a juicy future; and by the Faculty Research Development Fund of the University of Auckland.

## Contributions by the authors

R.J.S. and K.M.D. mined the genes, analysed the expression patterns, performed extraction for IAA measurements and co-wrote the paper. H.S.I. undertook the molecular experimentation. J.J.R. and T.J.L. measured the auxin concentrations.

## Conflict of interest statement

None declared.

## Supplementary Material

Additional Information
