# Supplementary material for: SEPALLATA1/2-suppressed mature apples have low ethylene, high auxin and reduced transcription of ripening-related genes
Source: AoB Plants. 2012 Dec 13;5:pls047. doi: 10.1093/aobpla/pls047 (PMC3551604; doi:10.1093/aobpla/pls047)
Supplement: Additional Information [file supp_5_pls047_index.html]

SEPALLATA1/2-suppressed mature apples have low ethylene, high auxin and reduced transcription of ripening-related genes — Additional Information 

# *SEPALLATA1/2*-suppressed mature apples have low ethylene, high auxin and reduced transcription of ripening-related genes

## Additional Information

**Files in this Data Supplement:**

- Additional Information 1 - pptx file
- Additional Information 2 - txt file
- Additional Information 3 - docx file
