# Supplementary material for: SEPALLATA1/2-suppressed mature apples have low ethylene, high auxin and reduced transcription of ripening-related genes
Source: AoB Plants. 2012 Dec 13;5:pls047. doi: 10.1093/aobpla/pls047 (PMC3551604; doi:10.1093/aobpla/pls047)
Supplement: Additional Information [file supp_pls047_pls047supp1.pptx]

## Slide 1
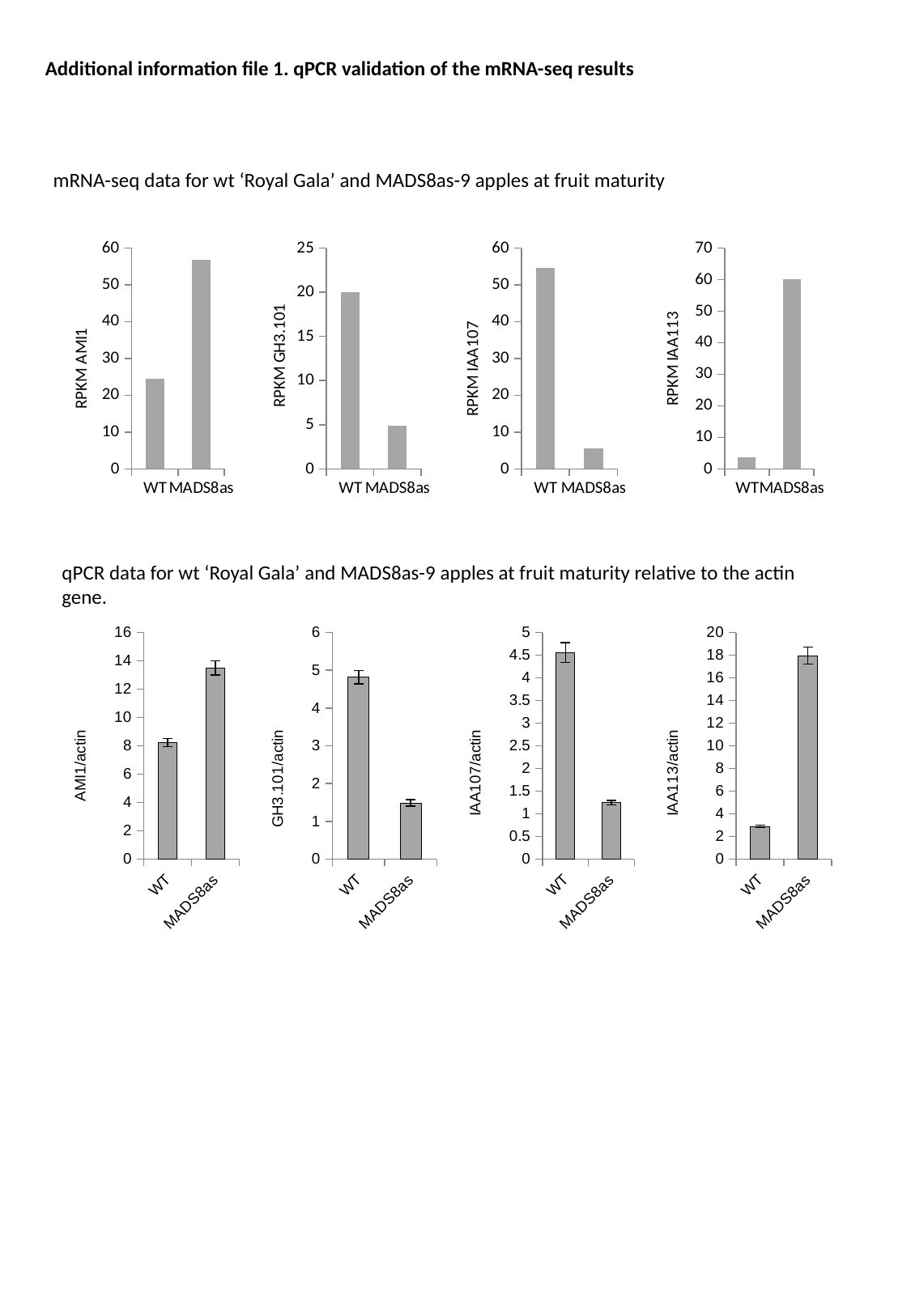

Additional information file 1. qPCR validation of the mRNA-seq results
mRNA-seq data for wt ‘Royal Gala’ and MADS8as-9 apples at fruit maturity
### Chart
| Category | |
|---|---|
| WT | 24.427694025684 |
| MADS8as | 56.8183842205194 |
### Chart
| Category | |
|---|---|
| WT | 19.964161894745 |
| MADS8as | 4.86056101383421 |
### Chart
| Category | |
|---|---|
| WT | 54.7005862646566 |
| MADS8as | 5.68406484776592 |
### Chart
| Category | |
|---|---|
| WT | 3.78235262333171 |
| MADS8as | 60.0772969042972 |qPCR data for wt ‘Royal Gala’ and MADS8as-9 apples at fruit maturity relative to the actin gene.
### Chart
| Category | |
|---|---|
| WT | 8.236 |
| MADS8as | 13.4985 |
### Chart
| Category | |
|---|---|
| WT | 4.81755 |
| MADS8as | 1.489575 |
### Chart
| Category | |
|---|---|
| WT | 4.5603 |
| MADS8as | 1.247625 |
### Chart
| Category | |
|---|---|
| WT | 2.8964499999999997 |
| MADS8as | 17.96625 |
