# Supplementary material for: SEPALLATA1/2-suppressed mature apples have low ethylene, high auxin and reduced transcription of ripening-related genes
Source: AoB Plants. 2012 Dec 13;5:pls047. doi: 10.1093/aobpla/pls047 (PMC3551604; doi:10.1093/aobpla/pls047)
Supplement: Additional Information [file supp_pls047_pls047supp3.docx]

| \| **Top Predominant RG expressors (10 fold)** \| \| \| \|  \| \|  \| \|  \| \|  \| \|  \| \|  \| \| \| --- \| --- \| --- \| --- \| --- \| --- \| --- \| --- \| --- \| --- \| --- \| --- \| --- \| --- \| --- \| --- \| \|  \| Gene Model \| MADS8as \| RG \| \|  \| \| Ratio \| \| Chr \| \| Position \| \| Function \| \| group \| \| \|  \| MDP0000195885 \| 2.008448 \| 4993.885 \| \|  \| \| 0.000602 \| \| chr10 \| \| 32.19915 \| \| 1-aminocyclopropane-1-carboxylate oxidase 1 \| \| Ethylene \| \| \|  \| MDP0000326734 \| 0.429486 \| 287.6439 \| \|  \| \| 0.004952 \| \| chr10 \| \| 18.13762 \| \| Polygalacturonase \| \| Cell wall \| \| \|  \| MDP0000182342 \| 1.132694 \| 389.2073 \| \|  \| \| 0.005466 \| \| chr1 \| \| 8.93019 \| \| Chavicol O-methyltransferase \| \| secondary metabolism \| \| \|  \| MDP0000219062 \| 1.029722 \| 367.5059 \| \|  \| \| 0.005508 \| \| chr1 \| \| 8.907618 \| \| Chavicol O-methyltransferase \| \| secondary metabolism \| \| \|  \| MDP0000200737 \| 5.614629 \| 912.5436 \| \|  \| \| 0.007241 \| \| chr5 \| \| 0.801646 \| \| 1-aminocyclopropane-1-carboxylate oxidase 2 \| \| Ethylene \| \| \|  \| MDP0000279839 \| 2.21727 \| 413.2814 \| \|  \| \| 0.007766 \| \| chr0 \| \| 112.3973 \| \| Chavicol O-methyltransferase \| \| secondary metabolism \| \| \|  \| MDP0000144836 \| 0 \| 106.7544 \| \|  \| \| 0.00928 \| \| chr16 \| \| 11.35099 \| \| Major allergen Mal d 1 \| \| allergen \| \| \|  \| MDP0000120188 \| 0 \| 66.17055 \| \|  \| \| 0.014887 \| \| chr15 \| \| 8.571195 \| \| unknown protein \| \|  \| \| \|  \| MDP0000288293 \| 1.729933 \| 137.6675 \| \|  \| \| 0.019687 \| \| chr16 \| \| 11.34038 \| \| Major allergen Pru ar 1 \| \| allergen \| \| \|  \| MDP0000294379 \| 0.790826 \| 67.5999 \| \|  \| \| 0.026105 \| \| chr16 \| \| 10.73767 \| \| Major allergen Mal d 1 \| \| allergen \| \| \|  \| MDP0000127542 \| 18.45586 \| 718.1329 \| \|  \| \| 0.027055 \| \| chr2 \| \| 6.055951 \| \| Beta-galactosidase \| \| cell wall \| \| \|  \| MDP0000209662 \| 4.421553 \| 195.4661 \| \|  \| \| 0.027595 \| \| chr16 \| \| 8.159903 \| \| Desiccation-related protein PCC13-62 \| \| dehydration \| \| \|  \| MDP0000188613 \| 7.200816 \| 295.1392 \| \|  \| \| 0.027692 \| \| chr16 \| \| 8.176406 \| \| Desiccation-related protein PCC13-62 \| \| dehydration \| \| \|  \| MDP0000366906 \| 20.13543 \| 754.696 \| \|  \| \| 0.027968 \| \| chr1 \| \| 25.15784 \| \| Os08g0545300 protein \| \|  \| \| \|  \| MDP0000300836 \| 2.656927 \| 128.4419 \| \|  \| \| 0.028251 \| \| chr15 \| \| 26.60918 \| \| Omega-hydroxypalmitate O-feruloyl transferase \| \| secondary metabolism \| \| \|  \| MDP0000908881 \| 0.349923 \| 45.95248 \| \|  \| \| 0.028751 \| \| chr16 \| \| 8.196742 \| \| Desiccation-related protein PCC13-62 \| \| dehydration \| \| \|  \| MDP0000151030 \| 0.812493 \| 61.37996 \| \|  \| \| 0.029056 \| \| chr15 \| \| 10.00981 \| \| Oleosin 18.5 kDa \| \| secondary metabolism \| \| \|  \| MDP0000874667 \| 22.28887 \| 775.331 \| \|  \| \| 0.029999 \| \| chr1 \| \| 25.15754 \| \| L-idonate 5-dehydrogenase \| \| secondary metabolism \| \| \|  \| MDP0000637737 \| 10.67997 \| 387.791 \| \|  \| \| 0.030042 \| \| chr2 \| \| 2.001341 \| \| Omega-hydroxypalmitate O-feruloyl transferase \| \| secondary metabolism \| \| \|  \| MDP0000199152 \| 0 \| 31.67096 \| \|  \| \| 0.030608 \| \| chr10 \| \| 30.04309 \| \| Myrcene synthase, chloroplastic \| \| secondary metabolism \| \| \|  \| MDP0000506359 \| 7.330694 \| 265.6071 \| \|  \| \| 0.031247 \| \| chr7 \| \| 23.41046 \| \| L-idonate 5-dehydrogenase \| \| secondary metabolism \| \| \|  \| MDP0000096349 \| 24.50448 \| 792.6959 \| \|  \| \| 0.032134 \| \| chr3 \| \| 32.27991 \| \| Glutathione S-transferase \| \|  \| \| \|  \| MDP0000185723 \| 4.826243 \| 176.6506 \| \|  \| \| 0.032796 \| \| chr2 \| \| 2.000277 \| \| Omega-hydroxypalmitate O-feruloyl transferase \| \| secondary metabolism \| \| \|  \| MDP0000546792 \| 2.806158 \| 112.9302 \| \|  \| \| 0.033408 \| \| chr6 \| \| 2.423856 \| \| Omega-hydroxypalmitate O-feruloyl transferase \| \| secondary metabolism \| \| \|  \| MDP0000666670 \| 0.216863 \| 35.29224 \| \|  \| \| 0.03353 \| \| chr17 \| \| 21.01939 \| \| Putative glutamine amidotransferase-like protein RP404 \| \| \| \| \|  \| MDP0000929213 \| 0.195105 \| 34.16204 \| \|  \| \| 0.033989 \| \| chr5 \| \| 7.44234 \| \| 9-cis-epoxycarotenoid dioxygenase NCED1, \| \| secondary metabolism \| \| \|  \| MDP0000260947 \| 1.968507 \| 86.29004 \| \|  \| \| 0.034007 \| \| chr15 \| \| 18.30522 \| \| Aldehyde dehydrogenase family 3 member F1 \| \| secondary metabolism \| \| \|  \| MDP0000407572 \| 1.200821 \| 61.49693 \| \|  \| \| 0.035215 \| \| chr6 \| \| 2.02845 \| \| Omega-hydroxypalmitate O-feruloyl transferase \| \| secondary metabolism \| \| \|  \| MDP0000942516 \| 169.5334 \| 4741.154 \| \|  \| \| 0.035961 \| \| chr16 \| \| 10.76744 \| \| Major allergen Mal d 1 \| \| allergen \| \| \|  \| MDP0000060934 \| 2.676378 \| 99.20417 \| \|  \| \| 0.036689 \| \| chr6 \| \| 2.066835 \| \| Omega-hydroxypalmitate O-feruloyl transferase \| \| secondary metabolism \| \| \|  \| MDP0000212045 \| 0.416224 \| 37.02724 \| \|  \| \| 0.037242 \| \| chr9 \| \| 29.28548 \| \| Protein RSI-1 \| \|  \| \| \|  \| MDP0000619822 \| 2.978007 \| 103.9017 \| \|  \| \| 0.037921 \| \| chr15 \| \| 26.61371 \| \| Omega-hydroxypalmitate O-feruloyl transferase \| \| secondary metabolism \| \| \|  \| MDP0000214714 \| 2.922619 \| 102.3232 \| \|  \| \| 0.037965 \| \| chr2 \| \| 1.950014 \| \| Omega-hydroxypalmitate O-feruloyl transferase \| \| secondary metabolism \| \| \|  \| MDP0000166457 \| 2.861543 \| 100.0066 \| \|  \| \| 0.038231 \| \| chr15 \| \| 26.60834 \| \| Omega-hydroxypalmitate O-feruloyl transferase \| \| secondary metabolism \| \| \|  \| MDP0000388537 \| 3.2305 \| 107.9745 \| \|  \| \| 0.038821 \| \| chr15 \| \| 26.61687 \| \| Omega-hydroxypalmitate O-feruloyl transferase \| \| secondary metabolism \| \| \|  \| MDP0000745371 \| 2.62515 \| 92.09255 \| \|  \| \| 0.038941 \| \| chr16 \| \| 8.143331 \| \| Desiccation-related protein PCC13-62 \| \| dehydration \| \| \|  \| MDP0000186091 \| 0.31258 \| 32.11049 \| \|  \| \| 0.039642 \| \| chr8 \| \| 1.726906 \| \| Jasmonate O-methyltransferase \| \| secondary metabolism \| \| \|  \| MDP0000612469 \| 0.261863 \| 30.78305 \| \|  \| \| 0.039702 \| \| chr16 \| \| 1.794944 \| \| Probable E3 ubiquitin-protein ligase RHA2B \| \|  \| \| \|  \| MDP0000428199 \| 2.664741 \| 91.03488 \| \|  \| \| 0.039819 \| \| chr13 \| \| 30.62545 \| \| Omega-hydroxypalmitate O-feruloyl transferase \| \| secondary metabolism \| \| \|  \| MDP0000323088 \| 2.318384 \| 81.84244 \| \|  \| \| 0.040057 \| \| chr2 \| \| 2.001109 \| \| Putative uncharacterized protein \| \|  \| \| \|  \| MDP0000183682 \| 0.205944 \| 28.78978 \| \|  \| \| 0.040482 \| \| chr11 \| \| 14.79227 \| \| Flavonol synthase/flavanone 3-hydroxylase \| \| secondary metabolism \| \| \|  \| MDP0000120347 \| 0.859594 \| 44.15192 \| \|  \| \| 0.041185 \| \| chr8 \| \| 15.3352 \| \| Flavonol synthase/flavanone 3-hydroxylase \| \| secondary metabolism \| \| \|  \| MDP0000305094 \| 0 \| 23.2139 \| \|  \| \| 0.041299 \| \| chr15 \| \| 8.555082 \| \| unknown protein \| \|  \| \| \|  \| MDP0000152370 \| 8.436982 \| 222.3064 \| \|  \| \| 0.04226 \| \| chr2 \| \| 2.001157 \| \| Omega-hydroxypalmitate O-feruloyl transferase \| \| secondary metabolism \| \| \|  \| MDP0000419297 \| 1.395576 \| 55.17785 \| \|  \| \| 0.042643 \| \| chr6 \| \| 2.603391 \| \| transferase family protein \| \|  \| \| \|  \| MDP0000563404 \| 1.801232 \| 64.31289 \| \|  \| \| 0.042889 \| \| chr6 \| \| 2.29158 \| \| Omega-hydroxypalmitate O-feruloyl transferase \| \| secondary metabolism \| \| \|  \| MDP0000266097 \| 12.81634 \| 316.2683 \| \|  \| \| 0.043548 \| \| chr3 \| \| 32.2858 \| \| Glutathione S-transferase \| \|  \| \| \|  \| MDP0000190508 \| 0.912492 \| 42.19817 \| \|  \| \| 0.044273 \| \| chr17 \| \| 22.79362 \| \| L-idonate 5-dehydrogenase \| \| secondary metabolism \| \| \|  \| MDP0000443072 \| 9.106486 \| 224.3541 \| \|  \| \| 0.044847 \| \| chr0 \| \| 4.318869 \| \| Omega-hydroxypalmitate O-feruloyl transferase \| \| secondary metabolism \| \| \|  \| MDP0000506825 \| 0.33298 \| 27.85859 \| \|  \| \| 0.04619 \| \| chr9 \| \| 14.38463 \| \| Indole-3-acetate beta-glucosyltransferase 2 \| \| secondary metabolism \| \| \|  \| MDP0000713234 \| 0.322348 \| 27.53805 \| \|  \| \| 0.046336 \| \| chr8 \| \| 1.726521 \| \| Jasmonate O-methyltransferase \| \| secondary metabolism \| \| \|  \| MDP0000186011 \| 14.77368 \| 337.7694 \| \|  \| \| 0.046562 \| \| chr5 \| \| 15.50313 \| \| Lipoxygenase 1 \| \| secondary metabolism \| \| \|  \| MDP0000123747 \| 0.080206 \| 21.82991 \| \|  \| \| 0.047315 \| \| chr6 \| \| 21.2948 \| \| 2-isopropylmalate synthase A \| \| secondary metabolism \| \| \|  \| MDP0000313382 \| 0.732247 \| 34.50896 \| \|  \| \| 0.048783 \| \| chr0 \| \| 13.67014 \| \| FPF1 (FLOWERING PROMOTING FACTOR 1) \| \| regulation \| \| \|  \| MDP0000152278 \| 0 \| 19.39846 \| \|  \| \| 0.049023 \| \| chr0 \| \| 3.234062 \| \| (+)-alpha-pinene synthase, chloroplastic \| \| secondary metabolism \| \| \|  \| MDP0000162146 \| 0.564876 \| 30.50969 \| \|  \| \| 0.049663 \| \| chr16 \| \| 8.191687 \| \| unknown protein \| \|  \| \| \|  \| MDP0000251339 \| 1.73808 \| 54.07072 \| \|  \| \| 0.049719 \| \| chr6 \| \| 2.580917 \| \| Omega-hydroxypalmitate O-feruloyl transferase \| \| secondary metabolism \| \| \|  \| MDP0000209689 \| 0.416224 \| 27.32963 \| \|  \| \| 0.049991 \| \| chr17 \| \| 22.51391 \| \| Protein RSI-1 \| \|  \| \| \|  \| MDP0000950619 \| 16.94628 \| 357.2283 \| \|  \| \| 0.050097 \| \| chr8 \| \| 0.680274 \| \| Probable cinnamyl alcohol dehydrogenase 6 \| \| secondary metabolism \| \| \|  \| MDP0000320017 \| 9.780539 \| 213.8194 \| \|  \| \| 0.050184 \| \| chr10 \| \| 17.69382 \| \| Probable xyloglucan endotransglucosylase/hydrolase protein 23 \| \| Cell wall \| \| \|  \| MDP0000772420 \| 8.608627 \| 187.4378 \| \|  \| \| 0.050991 \| \| chr1 \| \| 21.91264 \| \| Expansin-A8 \| \| Cell wall \| \| \|  \| MDP0000603942 \| 3.007368 \| 76.32057 \| \|  \| \| 0.051828 \| \| chr16 \| \| 1.538299 \| \| Leucoanthocyanidin reductase \| \| secondary metabolism \| \| \|  \| MDP0000311104 \| 8.21875 \| 175.8214 \| \|  \| \| 0.052136 \| \| chr0 \| \| 119.5793 \| \| transferase family protein \| \|  \| \| \|  \| MDP0000452083 \| 11.01508 \| 228.8227 \| \|  \| \| 0.05228 \| \| chr9 \| \| 4.308268 \| \| Lipoxygenase 1 \| \| secondary metabolism \| \| \|  \| MDP0000848959 \| 9.599627 \| 201.3923 \| \|  \| \| 0.052372 \| \| chr8 \| \| 7.736833 \| \| lactoylglutathione lyase family protein / glyoxalase I family protein \| \| secondary metabolism \| \| \|  \| MDP0000376284 \| 2.50614 \| 64.19028 \| \|  \| \| 0.053783 \| \| chr16 \| \| 1.530246 \| \| Leucoanthocyanidin reductase \| \| secondary metabolism \| \| \|  \| MDP0000180472 \| 1.757392 \| 50.01861 \| \|  \| \| 0.054047 \| \| chr2 \| \| 4.094874 \| \| UPF0497 membrane protein 8 \| \|  \| \| \|  \| MDP0000263664 \| 1.757392 \| 50.01861 \| \|  \| \| 0.054047 \| \| chr14 \| \| 20.47017 \| \| UPF0497 membrane protein 8 \| \|  \| \| \|  \| MDP0000260404 \| 1.029722 \| 36.53249 \| \|  \| \| 0.054079 \| \| chr8 \| \| 15.33339 \| \| Flavonol synthase/flavanone 3-hydroxylase \| \| secondary metabolism \| \| \|  \| MDP0000431696 \| 9.340469 \| 186.9584 \| \|  \| \| 0.055015 \| \| chr1 \| \| 21.92689 \| \| Expansin-A8 \| \| Cell wall \| \| \|  \| MDP0000264351 \| 24.08543 \| 449.0422 \| \|  \| \| 0.05574 \| \| chr7 \| \| 5.081985 \| \| Hyoscyamine 6-dioxygenase \| \| secondary metabolism \| \| \|  \| MDP0000825508 \| 1.658184 \| 46.19874 \| \|  \| \| 0.056319 \| \| chr13 \| \| 12.34522 \| \| Desiccation-related protein PCC13-62 \| \|  \| \| \|  \| MDP0000453295 \| 0.170437 \| 19.67452 \| \|  \| \| 0.056613 \| \| chr6 \| \| 21.36599 \| \| 2-isopropylmalate synthase A \| \| secondary metabolism \| \| \|  \| MDP0000171928 \| 1.984699 \| 50.84949 \| \|  \| \| 0.057565 \| \| chr16 \| \| 1.53604 \| \| Leucoanthocyanidin reductase \| \| secondary metabolism \| \| \|  \| MDP0000136671 \| 3.456409 \| 75.99185 \| \|  \| \| 0.057882 \| \| chr4 \| \| 19.34214 \| \| F-box protein PP2-B15 \| \| Regulation \| \| \|  \| MDP0000523205 \| 27.91822 \| 497.3233 \| \|  \| \| 0.058031 \| \| chr7 \| \| 5.077703 \| \| Hyoscyamine 6-dioxygenase \| \| secondary metabolism \| \| \|  \| MDP0000548790 \| 30.86597 \| 546.1294 \| \|  \| \| 0.058242 \| \| chr5 \| \| 15.49871 \| \| Lipoxygenase 1 \| \| secondary metabolism \| \| \|  \| MDP0000416548 \| 21.87737 \| 383.6921 \| \|  \| \| 0.059469 \| \| chr15 \| \| 14.72394 \| \| Beta-galactosidase \| \| Cell wall \| \| \|  \| MDP0000450991 \| 37.34712 \| 638.5008 \| \|  \| \| 0.059964 \| \| chr9 \| \| 4.301963 \| \| Lipoxygenase A \| \| secondary metabolism \| \| \|  \| MDP0000285427 \| 1.191004 \| 34.81262 \| \|  \| \| 0.06118 \| \| chr17 \| \| 22.78936 \| \| L-idonate 5-dehydrogenase \| \| secondary metabolism \| \| \|  \| MDP0000216907 \| 0.494267 \| 23.29355 \| \|  \| \| 0.061509 \| \| chr16 \| \| 10.80577 \| \| Major allergen Mal d 1 \| \| allergen \| \| \|  \| MDP0000422832 \| 13.94674 \| 234.7006 \| \|  \| \| 0.063414 \| \| chr8 \| \| 0.677195 \| \| Probable cinnamyl alcohol dehydrogenase 6 \| \| secondary metabolism \| \| \|  \| MDP0000386591 \| 0.395413 \| 20.93802 \| \|  \| \| 0.063607 \| \| chr10 \| \| 10.94742 \| \| Indole-3-acetic acid-induced protein ARG7 \| \| hormone \| \| \|  \| MDP0000860513 \| 0 \| 14.3575 \| \|  \| \| 0.065115 \| \| chr8 \| \| 28.53707 \| \| N/A \| \|  \| \| \|  \| MDP0000273257 \| 0.44304 \| 21.11397 \| \|  \| \| 0.065255 \| \| chr5 \| \| 9.3846 \| \| Zinc transporter 1 \| \|  \| \| \|  \| MDP0000229093 \| 0.514861 \| 21.91949 \| \|  \| \| 0.066095 \| \| chr14 \| \| 19.22001 \| \| Hyoscyamine 6-dioxygenase \| \| secondary metabolism \| \| \|  \| MDP0000440685 \| 0.818096 \| 25.99203 \| \|  \| \| 0.067357 \| \| chr3 \| \| 25.49185 \| \| N/A \| \|  \| \| \|  \| MDP0000877539 \| 9.705597 \| 153.7993 \| \|  \| \| 0.069158 \| \| chr15 \| \| 38.53105 \| \| Probable carotenoid cleavage dioxygenase 4, chloroplastic \| \| secondary metabolism \| \| \|  \| MDP0000243685 \| 0.718933 \| 23.79321 \| \|  \| \| 0.069331 \| \| chr12 \| \| 21.33051 \| \| Auxin-induced protein 10A5 \| \| hormone \| \| \|  \| MDP0000891556 \| 0 \| 13.34104 \| \|  \| \| 0.06973 \| \| chr0 \| \| 70.74554 \| \| Palmitoyl-monogalactosyldiacylglycerol delta-7 desaturase, chloroplastic \| \| secondary metabolism \| \| \|  \| MDP0000306389 \| 0.395413 \| 18.84422 \| \|  \| \| 0.070318 \| \| chr10 \| \| 10.99977 \| \| Indole-3-acetic acid-induced protein ARG7 \| \| hormone \| \| \|  \| MDP0000228056 \| 5.64876 \| 91.97775 \| \|  \| \| 0.071509 \| \| chr7 \| \| 5.147298 \| \| oxidoreductase, 2OG-Fe(II) oxygenase family protein \| \| \| \| \|  \| MDP0000288533 \| 10.03741 \| 148.5794 \| \|  \| \| 0.07379 \| \| chr10 \| \| 29.26616 \| \| Sugar carrier protein C \| \|  \| \| \|  \| MDP0000195633 \| 1.724544 \| 35.74466 \| \|  \| \| 0.074148 \| \| chr1 \| \| 28.76322 \| \| copper-binding family protein \| \|  \| \| \|  \| MDP0000236441 \| 1.724544 \| 35.74466 \| \|  \| \| 0.074148 \| \| chr1 \| \| 28.76307 \| \| copper-binding family protein \| \|  \| \| \|  \| MDP0000921871 \| 2.28123 \| 43.21823 \| \|  \| \| 0.074205 \| \| chr13 \| \| 1.740926 \| \| unknown protein \| \|  \| \| \|  \| MDP0000252292 \| 0 \| 12.40772 \| \|  \| \| 0.074584 \| \| chr17 \| \| 23.85055 \| \| Glutathione S-transferase \| \|  \| \| \|  \| MDP0000348422 \| 1.820317 \| 36.62815 \| \|  \| \| 0.074952 \| \| chr2 \| \| 28.29376 \| \| N/A \| \|  \| \| \|  \| MDP0000122086 \| 10.54435 \| 152.349 \| \|  \| \| 0.075282 \| \| chr17 \| \| 0.593567 \| \| Pteridine reductase 1 \| \| secondary metabolism \| \| \|  \| MDP0000651353 \| 2.28123 \| 42.14448 \| \|  \| \| 0.076052 \| \| chr3 \| \| 7.352263 \| \| unknown protein \| \|  \| \| \|  \| MDP0000150771 \| 0.444285 \| 17.87966 \| \|  \| \| 0.076499 \| \| chr8 \| \| 12.40442 \| \| Snakin-1 \| \|  \| \| \|  \| MDP0000320961 \| 0.261863 \| 15.25286 \| \|  \| \| 0.077639 \| \| chr3 \| \| 33.47046 \| \| unknown protein \| \|  \| \| \|  \| MDP0000767337 \| 0.261863 \| 15.25286 \| \|  \| \| 0.077639 \| \| chr1 \| \| 28.57439 \| \| unknown protein \| \|  \| \| \|  \| MDP0000816018 \| 0 \| 11.86488 \| \|  \| \| 0.077731 \| \| chr17 \| \| 8.407305 \| \| Palmitoyl-monogalactosyldiacylglycerol delta-7 desaturase, chloroplastic \| \| secondary metabolism \| \| \|  \| MDP0000891555 \| 0 \| 11.86488 \| \|  \| \| 0.077731 \| \| chr0 \| \| 70.74013 \| \| Palmitoyl-monogalactosyldiacylglycerol delta-7 desaturase, chloroplastic \| \| secondary metabolism \| \| \|  \| MDP0000463638 \| 3.056334 \| 51.18184 \| \|  \| \| 0.077735 \| \| chr11 \| \| 8.10998 \| \| Probable LRR receptor-like serine/threonine-protein kinase At3g47570 \| \| \| \| \|  \| MDP0000702760 \| 0 \| 11.81119 \| \|  \| \| 0.078057 \| \| chr16 \| \| 18.09291 \| \| GDP-mannose 4,6 dehydratase 2 \| \| secondary metabolism \| \| \|  \| MDP0000297328 \| 0 \| 11.76293 \| \|  \| \| 0.078352 \| \| chr3 \| \| 6.467601 \| \| Snakin-1 \| \|  \| \| \|  \| MDP0000195254 \| 21.76994 \| 286.0746 \| \|  \| \| 0.079317 \| \| chr15 \| \| 1.842484 \| \| Snakin-1 \| \|  \| \| \|  \| MDP0000116244 \| 0 \| 11.58834 \| \|  \| \| 0.079439 \| \| chr16 \| \| 11.4337 \| \| Major allergen Mal d 1 \| \| allergen \| \| \|  \| MDP0000175691 \| 0.873152 \| 22.32508 \| \|  \| \| 0.080306 \| \| chr5 \| \| 13.77415 \| \| 2'-deoxymugineic-acid 2'-dioxygenase \| \| secondary metabolism \| \| \|  \| MDP0000377710 \| 20.47992 \| 264.6829 \| \|  \| \| 0.080848 \| \| chr15 \| \| 38.40085 \| \| Omega-hydroxypalmitate O-feruloyl transferase \| \| secondary metabolism \| \| \|  \| MDP0000481065 \| 20.47992 \| 264.6829 \| \|  \| \| 0.080848 \| \| chr15 \| \| 38.37551 \| \| Omega-hydroxypalmitate O-feruloyl transferase \| \| secondary metabolism \| \| \|  \| MDP0000119926 \| 3.954132 \| 60.14923 \| \|  \| \| 0.081017 \| \| chr12 \| \| 25.11952 \| \| N/A \| \|  \| \| \|  \| MDP0000210067 \| 3.04164 \| 48.13956 \| \|  \| \| 0.082248 \| \| chr0 \| \| 3.170801 \| \| Putative uncharacterized protein \| \|  \| \| \|  \| MDP0000720497 \| 0.316331 \| 14.74037 \| \|  \| \| 0.083628 \| \| chr0 \| \| 106.3753 \| \| Indole-3-acetic acid-induced protein ARG7 \| \| hormone \| \| \|  \| MDP0000192132 \| 1.123333 \| 24.03114 \| \|  \| \| 0.084828 \| \| chr13 \| \| 1.316425 \| \| 21 kDa protein \| \|  \| \| \|  \| MDP0000250546 \| 5.572219 \| 76.26207 \| \|  \| \| 0.085064 \| \| chr1 \| \| 25.17313 \| \| L-idonate 5-dehydrogenase \| \| secondary metabolism \| \| \|  \| MDP0000636632 \| 8.031831 \| 103.288 \| \|  \| \| 0.086605 \| \| chr6 \| \| 17.347 \| \| F-box protein At1g67340 \| \| Regulation \| \| \|  \| MDP0000899351 \| 1.122624 \| 23.38189 \| \|  \| \| 0.087057 \| \| chr13 \| \| 16.30861 \| \| 2'-deoxymugineic-acid 2'-dioxygenase \| \| secondary metabolism \| \| \|  \| MDP0000189388 \| 3.954132 \| 55.39059 \| \|  \| \| 0.087854 \| \| chr12 \| \| 25.11448 \| \| N/A \| \|  \| \| \|  \| MDP0000231748 \| 3.58479 \| 50.50435 \| \|  \| \| 0.089018 \| \| chr17 \| \| 18.19038 \| \| AP2-like ethylene-responsive transcription factor At1g16060 \| \| Transcription factor \| \| \|  \| MDP0000744887 \| 15.91297 \| 188.4422 \| \|  \| \| 0.089278 \| \| chr15 \| \| 38.51909 \| \| Probable carotenoid cleavage dioxygenase 4, chloroplastic \| \| secondary metabolism \| \| \|  \| MDP0000150480 \| 0.670192 \| 17.46018 \| \|  \| \| 0.090475 \| \| chr16 \| \| 8.212106 \| \| Desiccation-related protein PCC13-62 \| \| dehydration \| \| \|  \| MDP0000140483 \| 33.87798 \| 382.1257 \| \|  \| \| 0.091035 \| \| chr16 \| \| 8.973341 \| \| Beta-D-xylosidase 1 \| \| Cell wall \| \| \|  \| MDP0000282945 \| 2.798468 \| 40.66861 \| \|  \| \| 0.091159 \| \| chr6 \| \| 2.07273 \| \| Probable galacturonosyltransferase-like 1 \| \|  \| \| \|  \| MDP0000423529 \| 15.02259 \| 173.3569 \| \|  \| \| 0.091895 \| \| chr9 \| \| 16.56066 \| \| Cytokinin-O-glucosyltransferase 1 \| \| secondary metabolism \| \| \|  \| MDP0000255777 \| 0.250262 \| 12.58932 \| \|  \| \| 0.092003 \| \| chr9 \| \| 4.360445 \| \| PI-PLC X domain-containing protein At5g67130 \| \|  \| \| \|  \| MDP0000200799 \| 0.537977 \| 15.38304 \| \|  \| \| 0.093876 \| \| chr16 \| \| 3.106786 \| \| ZF-HD homeobox protein At4g24660 \| \| Transcription factor \| \| \|  \| MDP0000294667 \| 0.118743 \| 10.81483 \| \|  \| \| 0.09469 \| \| chr8 \| \| 21.94473 \| \| Flavonol synthase/flavanone 3-hydroxylase \| \| secondary metabolism \| \| \|  \| MDP0000159240 \| 0 \| 9.558663 \| \|  \| \| 0.094709 \| \| chr5 \| \| 15.30734 \| \| Polygalacturonase \| \| Cell wall \| \| \|  \| MDP0000879058 \| 0.988533 \| 19.89112 \| \|  \| \| 0.095186 \| \| chr11 \| \| 28.06913 \| \| Uncharacterized aarF domain-containing protein kinase At4g31390, chloroplastic \| \| \| \| \|  \| MDP0000886270 \| 0 \| 9.481369 \| \|  \| \| 0.095407 \| \| chr0 \| \| 7.839952 \| \| Auxin-induced protein 15A \| \| hormone \| \| \|  \| MDP0000567466 \| 3.081142 \| 41.3322 \| \|  \| \| 0.096408 \| \| chr17 \| \| 24.37647 \| \| N/A \| \|  \| \| \|  \| MDP0000271244 \| 6.97788 \| 81.68292 \| \|  \| \| 0.096488 \| \| chr13 \| \| 1.773225 \| \| Probable ribose-5-phosphate isomerase \| \|  \| \| \|  \| MDP0000186964 \| 12.82829 \| 141.7712 \| \|  \| \| 0.096856 \| \| chr14 \| \| 23.33503 \| \| octicosapeptide/Phox/Bem1p (PB1) domain-containing protein \| \| \| \| \|  \| MDP0000391248 \| 0.103241 \| 10.38701 \| \|  \| \| 0.096886 \| \| chr2 \| \| 0.305764 \| \| Chavicol O-methyltransferase \| \| secondary metabolism \| \| \|  \| MDP0000651801 \| 0 \| 9.261049 \| \|  \| \| 0.097456 \| \| chr16 \| \| 12.50197 \| \| FPF1 (FLOWERING PROMOTING FACTOR 1) \| \| regulation \| \| \|  \| MDP0000295562 \| 5.735664 \| 68.1061 \| \|  \| \| 0.097468 \| \| chr11 \| \| 22.64987 \| \| Glucan endo-1,3-beta-glucosidase 13 \| \| cell wall \| \| \|  \| MDP0000291081 \| 2.388402 \| 33.72568 \| \|  \| \| 0.097576 \| \| chr12 \| \| 21.15197 \| \| Chaperone protein dnaJ 11, chloroplastic \| \| regulation \| \| \|  \| MDP0000292462 \| 0.484575 \| 14.16396 \| \|  \| \| 0.097902 \| \| chr17 \| \| 22.78068 \| \| L-idonate 5-dehydrogenase \| \| secondary metabolism \| \| \|  \| MDP0000759591 \| 5.822568 \| 68.56627 \| \|  \| \| 0.098073 \| \| chr11 \| \| 22.65312 \| \| Glucan endo-1,3-beta-glucosidase 13 \| \| cell wall \| \| \|  \| MDP0000906812 \| 0 \| 9.178312 \| \|  \| \| 0.098248 \| \| chr6 \| \| 4.983654 \| \| Expansin-like B1 \| \| cell wall \| \| \|  \| MDP0000874543 \| 0.782996 \| 16.99919 \| \|  \| \| 0.09906 \| \| chr0 \| \| 7.701292 \| \| Indole-3-acetic acid-induced protein ARG7 \| \| hormone \| \| \|  \| MDP0000199110 \| 0.734514 \| 16.46519 \| \|  \| \| 0.099313 \| \| chr16 \| \| 3.128389 \| \| ZF-HD homeobox protein At4g24660 \| \| Transcription factor \| \| \|  \| MDP0000532986 \| 0.734514 \| 16.46519 \| \|  \| \| 0.099313 \| \| chr16 \| \| 3.126785 \| \| ZF-HD homeobox protein At4g24660 \| \| Transcription factor \| \| \| **Top predominant MADS8as expressors (10 fold)** \| \| \| \| \| \| \| \|  \| \|  \| \|  \| \|  \| \| \|  \|  \| MADS8as \| RG \| \|  \| \| Ratio \| \|  \| \|  \| \|  \| \|  \| \| \|  \| MDP0000360414 \| 131.8458 \| 1.380529 \| \|  \| \| 55.80515 \| \| chr2 \| \| 12.50077 \| \| Dehydrin \| \| dehydration \| \| \|  \| MDP0000689622 \| 103.5606 \| 0.997049 \| \|  \| \| 52.35756 \| \| chr2 \| \| 12.50027 \| \| XERO1 (DEHYDRIN XERO 1) \| \| dehydration \| \| \|  \| MDP0000292266 \| 52.36553 \| 0.282946 \| \|  \| \| 41.59608 \| \| chr13 \| \| 3.244491 \| \| Probable E3 ubiquitin-protein ligase RHA2B \| \| regulation \| \| \|  \| MDP0000256575 \| 49.15948 \| 0.282946 \| \|  \| \| 39.0971 \| \| chr13 \| \| 3.232874 \| \| Probable E3 ubiquitin-protein ligase RHA2B \| \| regulation \| \| \|  \| MDP0000262784 \| 49.15948 \| 0.282946 \| \|  \| \| 39.0971 \| \| chr13 \| \| 3.235316 \| \| Probable E3 ubiquitin-protein ligase RHA2B \| \| regulation \| \| \|  \| MDP0000285931 \| 54.66684 \| 0.650502 \| \|  \| \| 33.72722 \| \| chr2 \| \| 7.040518 \| \| Flavonoid 3'-monooxygenase \| \| secondary metabolism \| \| \|  \| MDP0000175504 \| 85.08892 \| 1.59023 \| \|  \| \| 33.23602 \| \| chr0 \| \| 118.3379 \| \| Defensin-like protein \| \|  \| \| \|  \| MDP0000327289 \| 333.4295 \| 9.337227 \| \|  \| \| 32.35196 \| \| chr0 \| \| 23.77188 \| \| Uncharacterized protein TC_0114 \| \|  \| \| \|  \| MDP0000132436 \| 42.12814 \| 0.391365 \| \|  \| \| 30.997 \| \| chr11 \| \| 19.79282 \| \| Photosystem II 5 kDa protein, chloroplastic \| \| photosynthesis \| \| \|  \| MDP0000614624 \| 204.4085 \| 5.678108 \| \|  \| \| 30.75849 \| \| chr12 \| \| 26.56674 \| \| Uncharacterized protein TC_0114 \| \|  \| \| \|  \| MDP0000528246 \| 49.52262 \| 0.650502 \| \|  \| \| 30.61047 \| \| chr2 \| \| 7.052382 \| \| Flavonoid 3'-monooxygenase \| \| secondary metabolism \| \| \|  \| MDP0000135675 \| 39.54132 \| 0.391365 \| \|  \| \| 29.13781 \| \| chr11 \| \| 19.78526 \| \| Photosystem II 5 kDa protein, chloroplastic \| \| photosynthesis \| \| \|  \| MDP0000235841 \| 210.7753 \| 6.742753 \| \|  \| \| 27.35143 \| \| chr2 \| \| 36.4775 \| \| Non-specific lipid-transfer protein 8 \| \| photosynthesis \| \| \|  \| MDP0000908727 \| 25.58556 \| 0 \| \|  \| \| 26.58556 \| \| chr9 \| \| 4.877155 \| \| unknown protein \| \|  \| \| \|  \| MDP0000731480 \| 98.8533 \| 2.791736 \| \|  \| \| 26.33445 \| \| chr17 \| \| 20.0916 \| \| Ribulose bisphosphate carboxylase small chain, chloroplastic \| \| photosynthesis \| \| \|  \| MDP0000308997 \| 42.30537 \| 0.650502 \| \|  \| \| 26.2377 \| \| chr2 \| \| 7.009719 \| \| Flavonoid 3'-monooxygenase \| \| secondary metabolism \| \| \|  \| MDP0000398674 \| 56.65055 \| 1.207963 \| \|  \| \| 26.11029 \| \| chr0 \| \| 35.38608 \| \| Cytochrome P450 71D9 \| \| secondary metabolism \| \| \|  \| MDP0000524809 \| 96.87624 \| 2.791736 \| \|  \| \| 25.81304 \| \| chr17 \| \| 20.09153 \| \| Ribulose bisphosphate carboxylase small chain, chloroplastic \| \| photosynthesis \| \| \|  \| MDP0000316925 \| 196.7013 \| 6.742753 \| \|  \| \| 25.53372 \| \| chr0 \| \| 67.49294 \| \| Non-specific lipid-transfer protein 8 \| \|  \| \| \|  \| MDP0000199922 \| 38.08251 \| 0.569189 \| \|  \| \| 24.90619 \| \| chr2 \| \| 7.054732 \| \| Flavonoid 3'-monooxygenase \| \| secondary metabolism \| \| \|  \| MDP0000348963 \| 23.20904 \| 0 \| \|  \| \| 24.20904 \| \| chr5 \| \| 29.0643 \| \| N/A \| \|  \| \| \|  \| MDP0000480390 \| 6405.071 \| 263.7861 \| \|  \| \| 24.19338 \| \| chr0 \| \| 102.9433 \| \| Uncharacterized protein ART2 \| \|  \| \| \|  \| MDP0000047048 \| 623.4655 \| 24.83347 \| \|  \| \| 24.17273 \| \| chr0 \| \| 5.602277 \| \| Uncharacterized protein ART2 \| \|  \| \| \|  \| MDP0000392063 \| 7046.139 \| 294.781 \| \|  \| \| 23.82553 \| \| chr0 \| \| 107.0464 \| \| Uncharacterized protein ART2 \| \|  \| \| \|  \| MDP0000473113 \| 7046.139 \| 294.781 \| \|  \| \| 23.82553 \| \| chr0 \| \| 97.50822 \| \| Uncharacterized protein ART2 \| \|  \| \| \|  \| MDP0000647697 \| 7046.139 \| 294.781 \| \|  \| \| 23.82553 \| \| chr7 \| \| 21.63341 \| \| Uncharacterized protein ART2 \| \|  \| \| \|  \| MDP0000377780 \| 7046.139 \| 294.781 \| \|  \| \| 23.82553 \| \| chr0 \| \| 88.67265 \| \| Uncharacterized protein ART2 \| \|  \| \| \|  \| MDP0000555310 \| 7046.139 \| 294.781 \| \|  \| \| 23.82553 \| \| chr0 \| \| 89.62091 \| \| Uncharacterized protein ART2 \| \|  \| \| \|  \| MDP0000841405 \| 22.73626 \| 0 \| \|  \| \| 23.73626 \| \| chr13 \| \| 28.93295 \| \| Calcineurin B-like protein 9 \| \|  \| \| \|  \| MDP0000479393 \| 6646.055 \| 282.5809 \| \|  \| \| 23.43972 \| \| chr0 \| \| 123.9515 \| \| Uncharacterized protein ART2 \| \|  \| \| \|  \| MDP0000735656 \| 6535.526 \| 278.6241 \| \|  \| \| 23.37612 \| \| chr7 \| \| 6.740354 \| \| Uncharacterized protein ART2 \| \|  \| \| \|  \| MDP0000380188 \| 6558.878 \| 280.2728 \| \|  \| \| 23.32212 \| \| chr2 \| \| 11.13813 \| \| Uncharacterized protein ART2 \| \|  \| \| \|  \| MDP0000647699 \| 4685.846 \| 207.6883 \| \|  \| \| 22.4586 \| \| chr7 \| \| 21.63412 \| \| Putative uncharacterized protein Sb1138s002030 \| \|  \| \| \|  \| MDP0000310546 \| 47.60314 \| 1.219691 \| \|  \| \| 21.89636 \| \| chr0 \| \| 45.18448 \| \| Flavonoid 3'-monooxygenase \| \| secondary metabolism \| \| \|  \| MDP0000698024 \| 510.6137 \| 22.84148 \| \|  \| \| 21.45897 \| \| chr2 \| \| 12.48446 \| \| Dehydrin \| \| dehydration \| \| \|  \| MDP0000387469 \| 442.3621 \| 20.14533 \| \|  \| \| 20.96737 \| \| chr3 \| \| 1.165829 \| \| Transposon Ty3-I Gag-Pol polyprotein \| \|  \| \| \|  \| MDP0000269469 \| 57.70895 \| 1.915329 \| \|  \| \| 20.13802 \| \| chr2 \| \| 7.010482 \| \| Cytochrome P450 71A1 \| \| secondary metabolism \| \| \|  \| MDP0000445165 \| 4337.891 \| 221.0614 \| \|  \| \| 19.53915 \| \| chr0 \| \| 122.7996 \| \| Predicted protein \| \|  \| \| \|  \| MDP0000144167 \| 18.45262 \| 0 \| \|  \| \| 19.45262 \| \| chr1 \| \| 20.08612 \| \| Putative uncharacterized protein \| \|  \| \| \|  \| MDP0000217215 \| 122.9376 \| 5.71037 \| \|  \| \| 18.46956 \| \| chr10 \| \| 26.12015 \| \| Chlorophyll a-b binding protein 21, chloroplastic \| \| photosynthesis \| \| \|  \| MDP0000862169 \| 120.778 \| 5.702964 \| \|  \| \| 18.16778 \| \| chr2 \| \| 12.5039 \| \| Dehydrin \| \| dehydration \| \| \|  \| MDP0000360295 \| 15.93456 \| 0 \| \|  \| \| 16.93456 \| \| chr9 \| \| 4.87794 \| \| unknown protein \| \|  \| \| \|  \| MDP0000254705 \| 15.81653 \| 0 \| \|  \| \| 16.81653 \| \| chr14 \| \| 29.00349 \| \| Putative phytosulfokines 4 \| \| secondary metabolism \| \| \|  \| MDP0000208137 \| 17.21769 \| 0.125754 \| \|  \| \| 16.18266 \| \| chr4 \| \| 18.78868 \| \| Glycogenin-2 \| \|  \| \| \|  \| MDP0000231923 \| 17.10923 \| 0.134218 \| \|  \| \| 15.96626 \| \| chr4 \| \| 18.79083 \| \| Glycogenin-2 \| \|  \| \| \|  \| MDP0000133105 \| 51.78863 \| 2.347035 \| \|  \| \| 15.77176 \| \| chr15 \| \| 5.313437 \| \| Gibberellin 20 oxidase 1 \| \| hormone \| \| \|  \| MDP0000256812 \| 29.02256 \| 0.975753 \| \|  \| \| 15.19551 \| \| chr2 \| \| 7.062474 \| \| Flavonoid 3'-monooxygenase \| \| secondary metabolism \| \| \|  \| MDP0000249827 \| 20.18471 \| 0.438493 \| \|  \| \| 14.72702 \| \| chr2 \| \| 15.02415 \| \| Dof zinc finger protein DOF5.3 \| \| Transcription factor \| \| \|  \| MDP0000164386 \| 21.38713 \| 0.526743 \| \|  \| \| 14.66333 \| \| chr15 \| \| 8.812838 \| \| Whole genome shotgun sequence of line PN40024, scaffold_118.assembly12x \| \| \| \| \|  \| MDP0000185950 \| 48.64436 \| 2.410132 \| \|  \| \| 14.5579 \| \| chr10 \| \| 8.101574 \| \| N/A \| \|  \| \| \|  \| MDP0000427722 \| 17.4598 \| 0.271922 \| \|  \| \| 14.51331 \| \| chr16 \| \| 4.042599 \| \| MLP-like protein 423 \| \|  \| \| \|  \| MDP0000134685 \| 13.46088 \| 0 \| \|  \| \| 14.46088 \| \| chr1 \| \| 7.13243 \| \| Serine hydroxymethyltransferase, mitochondrial \| \|  \| \| \|  \| MDP0000148685 \| 22.84382 \| 0.650923 \| \|  \| \| 14.44272 \| \| chr2 \| \| 26.31535 \| \| Probable receptor-like protein kinase At5g39030 \| \| regulation \| \| \|  \| MDP0000362305 \| 21.23835 \| 0.548595 \| \|  \| \| 14.36034 \| \| chr16 \| \| 7.487744 \| \| Defensin-like protein \| \| defence \| \| \|  \| MDP0000126259 \| 17.06235 \| 0.286822 \| \|  \| \| 14.0364 \| \| chr9 \| \| 0.695589 \| \| Flowering locus C-like gene \| \| regulation \| \| \|  \| MDP0000233682 \| 12.94716 \| 0 \| \|  \| \| 13.94716 \| \| chr17 \| \| 9.596227 \| \| Whole genome shotgun sequence of line PN40024, scaffold_57.assembly12x \| \| \| \| \|  \| MDP0000574183 \| 23.48515 \| 0.761383 \| \|  \| \| 13.9011 \| \| chr0 \| \| 9.388448 \| \| Probable receptor-like protein kinase At5g39020 \| \| regulation \| \| \|  \| MDP0000265684 \| 37.93829 \| 1.810856 \| \|  \| \| 13.85282 \| \| chr5 \| \| 13.74001 \| \| Peroxisomal (S)-2-hydroxy-acid oxidase \| \|  \| \| \|  \| MDP0000614622 \| 144.9848 \| 9.550677 \| \|  \| \| 13.83654 \| \| chr12 \| \| 26.56551 \| \| Uncharacterized protein ORF91 \| \|  \| \| \|  \| MDP0000376384 \| 144.9848 \| 9.550677 \| \|  \| \| 13.83654 \| \| chr2 \| \| 36.07027 \| \| Uncharacterized protein ORF91 \| \|  \| \| \|  \| MDP0000366022 \| 713.9376 \| 51.44613 \| \|  \| \| 13.63185 \| \| chr17 \| \| 5.046844 \| \| Developmental protein SEPALLATA 1 \| \| transcription factor \| \| \|  \| MDP0000344696 \| 12.57305 \| 0 \| \|  \| \| 13.57305 \| \| chr10 \| \| 6.296615 \| \| N/A \| \|  \| \| \|  \| MDP0000289026 \| 17.27856 \| 0.3519 \| \|  \| \| 13.52065 \| \| chr1 \| \| 1.79154 \| \| unknown protein \| \|  \| \| \|  \| MDP0000320910 \| 54.45405 \| 3.110792 \| \|  \| \| 13.48987 \| \| chr12 \| \| 30.59802 \| \| LSH1 (LIGHT-DEPENDENT SHORT HYPOCOTYLS 1) \| \| regulation \| \| \|  \| MDP0000197775 \| 51.59402 \| 2.911169 \| \|  \| \| 13.44714 \| \| chr5 \| \| 0.584713 \| \| Early nodulin-like protein 2 \| \|  \| \| \|  \| MDP0000631333 \| 21.56799 \| 0.692166 \| \|  \| \| 13.33675 \| \| chr3 \| \| 23.47483 \| \| Putative uncharacterized protein \| \|  \| \| \|  \| MDP0000873376 \| 15.17532 \| 0.226357 \| \|  \| \| 13.18973 \| \| chr5 \| \| 27.22469 \| \| Blue copper protein \| \|  \| \| \|  \| MDP0000224488 \| 26.36088 \| 1.08769 \| \|  \| \| 13.10582 \| \| chr14 \| \| 28.88998 \| \| Germin-like protein subfamily 1 member 7 \| \|  \| \| \|  \| MDP0000668095 \| 41.94438 \| 2.313594 \| \|  \| \| 12.96006 \| \| chr0 \| \| 2.846566 \| \| AWPM-19-like membrane family protein \| \|  \| \| \|  \| MDP0000164095 \| 60.0773 \| 3.782353 \| \|  \| \| 12.77139 \| \| chr9 \| \| 18.98959 \| \| Auxin-responsive protein IAA26 \| \| hormone \| \| \|  \| MDP0000178782 \| 17.25837 \| 0.462719 \| \|  \| \| 12.48249 \| \| chr8 \| \| 25.16521 \| \| Transcription factor bHLH93 \| \| transcription factor \| \| \|  \| MDP0000735652 \| 4088.096 \| 327.0368 \| \|  \| \| 12.46536 \| \| chr7 \| \| 6.737624 \| \| Putative uncharacterized protein Sb0016s002240 \| \|  \| \| \|  \| MDP0000909197 \| 65.4477 \| 4.332005 \| \|  \| \| 12.46205 \| \| chr9 \| \| 19.99641 \| \| Photosystem I reaction center subunit VI-2, chloroplastic \| \| photosynthesis \| \| \|  \| MDP0000835914 \| 13.70417 \| 0.184883 \| \|  \| \| 12.4098 \| \| chr6 \| \| 0.89991 \| \| Glyceraldehyde-3-phosphate dehydrogenase B, chloroplastic \| \| photosynthesis \| \| \|  \| MDP0000175282 \| 17.14914 \| 0.462719 \| \|  \| \| 12.40781 \| \| chr8 \| \| 25.16522 \| \| Transcription factor bHLH93 \| \| transcription factor \| \| \|  \| MDP0000647701 \| 3693.326 \| 302.0359 \| \|  \| \| 12.19105 \| \| chr7 \| \| 21.63477 \| \| Putative uncharacterized protein Sb0016s002240 \| \|  \| \| \|  \| MDP0000500042 \| 3673.74 \| 302.1337 \| \|  \| \| 12.1225 \| \| chr0 \| \| 81.72679 \| \| Putative uncharacterized protein Sb0016s002240 \| \|  \| \| \|  \| MDP0000706975 \| 66.57903 \| 4.640319 \| \|  \| \| 11.98142 \| \| chr10 \| \| 20.18676 \| \| Peroxisomal (S)-2-hydroxy-acid oxidase \| \|  \| \| \|  \| MDP0000517479 \| 29.98394 \| 1.587717 \| \|  \| \| 11.97346 \| \| chr15 \| \| 13.64632 \| \| unknown protein \| \|  \| \| \|  \| MDP0000833352 \| 29.98394 \| 1.587717 \| \|  \| \| 11.97346 \| \| chr15 \| \| 13.64638 \| \| unknown protein \| \|  \| \| \|  \| MDP0000441919 \| 2343.82 \| 195.1173 \| \|  \| \| 11.95621 \| \| chr6 \| \| 2.96732 \| \| Putative uncharacterized protein Sb0016s002240 \| \|  \| \| \|  \| MDP0000866655 \| 37.55003 \| 2.259499 \| \|  \| \| 11.82698 \| \| chr9 \| \| 9.796199 \| \| Chlorophyll a-b binding protein 3, chloroplastic \| \| photosynthesis \| \| \|  \| MDP0000242247 \| 77.50854 \| 5.667936 \| \|  \| \| 11.77404 \| \| chr0 \| \| 8.30883 \| \| Probable fructose-bisphosphate aldolase 2, chloroplastic \| \| photosynthesis \| \| \|  \| MDP0000203502 \| 14.828 \| 0.373893 \| \|  \| \| 11.52054 \| \| chr12 \| \| 21.10968 \| \| unknown protein \| \|  \| \| \|  \| MDP0000265874 \| 8962.423 \| 780.2976 \| \|  \| \| 11.47248 \| \| chr2 \| \| 12.70191 \| \| Dehydrin DHN1 \| \| dehydration \| \| \|  \| MDP0000605482 \| 264.9268 \| 22.61307 \| \|  \| \| 11.26185 \| \| chr9 \| \| 4.411551 \| \| Developmental protein SEPALLATA 1 \| \| transcription factor \| \| \|  \| MDP0000261585 \| 254.2657 \| 21.73314 \| \|  \| \| 11.22879 \| \| chr16 \| \| 7.489678 \| \| Defensin SD2 \| \|  \| \| \|  \| MDP0000790788 \| 10.13042 \| 0 \| \|  \| \| 11.13042 \| \| chr9 \| \| 19.61983 \| \| Dehydration-responsive element-binding protein 3 \| \| dehydration \| \| \|  \| MDP0000140596 \| 9.88533 \| 0 \| \|  \| \| 10.88533 \| \| chr2 \| \| 34.65389 \| \| Defensin-like protein 2 \| \|  \| \| \|  \| MDP0000203619 \| 9.88533 \| 0 \| \|  \| \| 10.88533 \| \| chr0 \| \| 99.10676 \| \| Defensin-like protein 2 \| \|  \| \| \|  \| MDP0000204533 \| 9.88533 \| 0 \| \|  \| \| 10.88533 \| \| chr2 \| \| 34.65439 \| \| Defensin-like protein 2 \| \|  \| \| \|  \| MDP0000216636 \| 9.88533 \| 0 \| \|  \| \| 10.88533 \| \| chr0 \| \| 102.8071 \| \| Defensin-like protein 2 \| \|  \| \| \|  \| MDP0000817900 \| 31.19371 \| 2.016254 \| \|  \| \| 10.67341 \| \| chr13 \| \| 29.26558 \| \| Zinc finger protein MAGPIE \| \|  \| \| \|  \| MDP0000151095 \| 80.7625 \| 6.705641 \| \|  \| \| 10.61073 \| \| chr1 \| \| 14.17805 \| \| Chlorophyll a-b binding protein 6A, chloroplastic \| \| photosynthesis \| \| \|  \| MDP0000269859 \| 43.81276 \| 3.231176 \| \|  \| \| 10.59109 \| \| chr9 \| \| 9.826701 \| \| Chlorophyll a-b binding protein 3, chloroplastic \| \| photosynthesis \| \| \|  \| MDP0000266176 \| 12.96612 \| 0.340456 \| \|  \| \| 10.41894 \| \| chr6 \| \| 21.08131 \| \| GDSL esterase/lipase At1g74460 \| \| secondary metabolism \| \| \|  \| MDP0000142895 \| 47.54014 \| 3.676142 \| \|  \| \| 10.38038 \| \| chr17 \| \| 16.0275 \| \| Photosystem I reaction center subunit VI-2, chloroplastic \| \| photosynthesis \| \| \|  \| MDP0000774924 \| 10.10277 \| 0.070856 \| \|  \| \| 10.36813 \| \| chr0 \| \| 53.68664 \| \| Probable carotenoid cleavage dioxygenase 4, chloroplastic \| \| secondary metabolism \| \| \|  \| MDP0000212688 \| 9.336145 \| 0 \| \|  \| \| 10.33615 \| \| chr6 \| \| 1.36193 \| \| Lupeol synthase 5 \| \| secondary metabolism \| \| \|  \| MDP0000508777 \| 174.1629 \| 15.98322 \| \|  \| \| 10.31388 \| \| chr17 \| \| 5.034003 \| \| Developmental protein SEPALLATA 1 \| \| transcription factor \| \| \|  \| MDP0000259706 \| 53.25974 \| 4.273066 \| \|  \| \| 10.28998 \| \| chr8 \| \| 6.556172 \| \| Chlorophyll a-b binding protein 6A, chloroplastic \| \| photosynthesis \| \| \|  \| MDP0000296410 \| 73.25008 \| 6.358619 \| \|  \| \| 10.09022 \| \| chr4 \| \| 1.100936 \| \| Alcohol dehydrogenase 1 \| \| secondary metabolism \| \| \|  \| MDP0000661382 \| 2964.392 \| 293.452 \| \|  \| \| 10.07088 \| \| chr13 \| \| 18.5856 \| \| Putative uncharacterized protein \| \|  \| \| \|  \| MDP0000659245 \| 16.84167 \| 0.775482 \| \|  \| \| 10.04892 \| \| chr11 \| \| 31.64682 \| \| 37 kDa inner envelope membrane protein, chloroplastic \| \| photosynthesis \| \| \|  \| MDP0000127549 \| 30.30832 \| 2.125688 \| \|  \| \| 10.01646 \| \| chr7 \| \| 23.65328 \| \| 3-hexulose-6-phosphate isomerase \| \| photosynthesis \| \| | | | |  |  |  |  |  |  |  |  |  |  |  |  |
| --- | --- | --- | --- | --- | --- | --- | --- | --- | --- | --- | --- | --- | --- | --- | --- | --- | --- | --- | --- | --- | --- | --- | --- | --- | --- | --- | --- | --- | --- | --- | --- | --- | --- | --- | --- | --- | --- | --- | --- | --- | --- | --- | --- | --- | --- | --- | --- | --- | --- | --- | --- | --- | --- | --- | --- | --- | --- | --- | --- | --- | --- | --- | --- | --- | --- | --- | --- | --- | --- | --- | --- | --- | --- | --- | --- | --- | --- | --- | --- | --- | --- | --- | --- | --- | --- | --- | --- | --- | --- | --- | --- | --- | --- | --- | --- | --- | --- | --- | --- | --- | --- | --- | --- | --- | --- | --- | --- | --- | --- | --- | --- | --- | --- | --- | --- | --- | --- | --- | --- | --- | --- | --- | --- | --- | --- | --- | --- | --- | --- | --- | --- | --- | --- | --- | --- | --- | --- | --- | --- | --- | --- | --- | --- | --- | --- | --- | --- | --- | --- | --- | --- | --- | --- | --- | --- | --- | --- | --- | --- | --- | --- | --- | --- | --- | --- | --- | --- | --- | --- | --- | --- | --- | --- | --- | --- | --- | --- | --- | --- | --- | --- | --- | --- | --- | --- | --- | --- | --- | --- | --- | --- | --- | --- | --- | --- | --- | --- | --- | --- | --- | --- | --- | --- | --- | --- | --- | --- | --- | --- | --- | --- | --- | --- | --- | --- | --- | --- | --- | --- | --- | --- | --- | --- | --- | --- | --- | --- | --- | --- | --- | --- | --- | --- | --- | --- | --- | --- | --- | --- | --- | --- | --- | --- | --- | --- | --- | --- | --- | --- | --- | --- | --- | --- | --- | --- | --- | --- | --- | --- | --- | --- | --- | --- | --- | --- | --- | --- | --- | --- | --- | --- | --- | --- | --- | --- | --- | --- | --- | --- | --- | --- | --- | --- | --- | --- | --- | --- | --- | --- | --- | --- | --- | --- | --- | --- | --- | --- | --- | --- | --- | --- | --- | --- | --- | --- | --- | --- | --- | --- | --- | --- | --- | --- | --- | --- | --- | --- | --- | --- | --- | --- | --- | --- | --- | --- | --- | --- | --- | --- | --- | --- | --- | --- | --- | --- | --- | --- | --- | --- | --- | --- | --- | --- | --- | --- | --- | --- | --- | --- | --- | --- | --- | --- | --- | --- | --- | --- | --- | --- | --- | --- | --- | --- | --- | --- | --- | --- | --- | --- | --- | --- | --- | --- | --- | --- | --- | --- | --- | --- | --- | --- | --- | --- | --- | --- | --- | --- | --- | --- | --- | --- | --- | --- | --- | --- | --- | --- | --- | --- | --- | --- | --- | --- | --- | --- | --- | --- | --- | --- | --- | --- | --- | --- | --- | --- | --- | --- | --- | --- | --- | --- | --- | --- | --- | --- | --- | --- | --- | --- | --- | --- | --- | --- | --- | --- | --- | --- | --- | --- | --- | --- | --- | --- | --- | --- | --- | --- | --- | --- | --- | --- | --- | --- | --- | --- | --- | --- | --- | --- | --- | --- | --- | --- | --- | --- | --- | --- | --- | --- | --- | --- | --- | --- | --- | --- | --- | --- | --- | --- | --- | --- | --- | --- | --- | --- | --- | --- | --- | --- | --- | --- | --- | --- | --- | --- | --- | --- | --- | --- | --- | --- | --- | --- | --- | --- | --- | --- | --- | --- | --- | --- | --- | --- | --- | --- | --- | --- | --- | --- | --- | --- | --- | --- | --- | --- | --- | --- | --- | --- | --- | --- | --- | --- | --- | --- | --- | --- | --- | --- | --- | --- | --- | --- | --- | --- | --- | --- | --- | --- | --- | --- | --- | --- | --- | --- | --- | --- | --- | --- | --- | --- | --- | --- | --- | --- | --- | --- | --- | --- | --- | --- | --- | --- | --- | --- | --- | --- | --- | --- | --- | --- | --- | --- | --- | --- | --- | --- | --- | --- | --- | --- | --- | --- | --- | --- | --- | --- | --- | --- | --- | --- | --- | --- | --- | --- | --- | --- | --- | --- | --- | --- | --- | --- | --- | --- | --- | --- | --- | --- | --- | --- | --- | --- | --- | --- | --- | --- | --- | --- | --- | --- | --- | --- | --- | --- | --- | --- | --- | --- | --- | --- | --- | --- | --- | --- | --- | --- | --- | --- | --- | --- | --- | --- | --- | --- | --- | --- | --- | --- | --- | --- | --- | --- | --- | --- | --- | --- | --- | --- | --- | --- | --- | --- | --- | --- | --- | --- | --- | --- | --- | --- | --- | --- | --- | --- | --- | --- | --- | --- | --- | --- | --- | --- | --- | --- | --- | --- | --- | --- | --- | --- | --- | --- | --- | --- | --- | --- | --- | --- | --- | --- | --- | --- | --- | --- | --- | --- | --- | --- | --- | --- | --- | --- | --- | --- | --- | --- | --- | --- | --- | --- | --- | --- | --- | --- | --- | --- | --- | --- | --- | --- | --- | --- | --- | --- | --- | --- | --- | --- | --- | --- | --- | --- | --- | --- | --- | --- | --- | --- | --- | --- | --- | --- | --- | --- | --- | --- | --- | --- | --- | --- | --- | --- | --- | --- | --- | --- | --- | --- | --- | --- | --- | --- | --- | --- | --- | --- | --- | --- | --- | --- | --- | --- | --- | --- | --- | --- | --- | --- | --- | --- | --- | --- | --- | --- | --- | --- | --- | --- | --- | --- | --- | --- | --- | --- | --- | --- | --- | --- | --- | --- | --- | --- | --- | --- | --- | --- | --- | --- | --- | --- | --- | --- | --- | --- | --- | --- | --- | --- | --- | --- | --- | --- | --- | --- | --- | --- | --- | --- | --- | --- | --- | --- | --- | --- | --- | --- | --- | --- | --- | --- | --- | --- | --- | --- | --- | --- | --- | --- | --- | --- | --- | --- | --- | --- | --- | --- | --- | --- | --- | --- | --- | --- | --- | --- | --- | --- | --- | --- | --- | --- | --- | --- | --- | --- | --- | --- | --- | --- | --- | --- | --- | --- | --- | --- | --- | --- | --- | --- | --- | --- | --- | --- | --- | --- | --- | --- | --- | --- | --- | --- | --- | --- | --- | --- | --- | --- | --- | --- | --- | --- | --- | --- | --- | --- | --- | --- | --- | --- | --- | --- | --- | --- | --- | --- | --- | --- | --- | --- | --- | --- | --- | --- | --- | --- | --- | --- | --- | --- | --- | --- | --- | --- | --- | --- | --- | --- | --- | --- | --- | --- | --- | --- | --- | --- | --- | --- | --- | --- | --- | --- | --- | --- | --- | --- | --- | --- | --- | --- | --- | --- | --- | --- | --- | --- | --- | --- | --- | --- | --- | --- | --- | --- | --- | --- | --- | --- | --- | --- | --- | --- | --- | --- | --- | --- | --- | --- | --- | --- | --- | --- | --- | --- | --- | --- | --- | --- | --- | --- | --- | --- | --- | --- | --- | --- | --- | --- | --- | --- | --- | --- | --- | --- | --- | --- | --- | --- | --- | --- | --- | --- | --- | --- | --- | --- | --- | --- | --- | --- | --- | --- | --- | --- | --- | --- | --- | --- | --- | --- | --- | --- | --- | --- | --- | --- | --- | --- | --- | --- | --- | --- | --- | --- | --- | --- | --- | --- | --- | --- | --- | --- | --- | --- | --- | --- | --- | --- | --- | --- | --- | --- | --- | --- | --- | --- | --- | --- | --- | --- | --- | --- | --- | --- | --- | --- | --- | --- | --- | --- | --- | --- | --- | --- | --- | --- | --- | --- | --- | --- | --- | --- | --- | --- | --- | --- | --- | --- | --- | --- | --- | --- | --- | --- | --- | --- | --- | --- | --- | --- | --- | --- | --- | --- | --- | --- | --- | --- | --- | --- | --- | --- | --- | --- | --- | --- | --- | --- | --- | --- | --- | --- | --- | --- | --- | --- | --- | --- | --- | --- | --- | --- | --- | --- | --- | --- | --- | --- | --- | --- | --- | --- | --- | --- | --- | --- | --- | --- | --- | --- | --- | --- | --- | --- | --- | --- | --- | --- | --- | --- | --- | --- | --- | --- | --- | --- | --- | --- | --- | --- | --- | --- | --- | --- | --- | --- | --- | --- | --- | --- | --- | --- | --- | --- | --- | --- | --- | --- | --- | --- | --- | --- | --- | --- | --- | --- | --- | --- | --- | --- | --- | --- | --- | --- | --- | --- | --- | --- | --- | --- | --- | --- | --- | --- | --- | --- | --- | --- | --- | --- | --- | --- | --- | --- | --- | --- | --- | --- | --- | --- | --- | --- | --- | --- | --- | --- | --- | --- | --- | --- | --- | --- | --- | --- | --- | --- | --- | --- | --- | --- | --- | --- | --- | --- | --- | --- | --- | --- | --- | --- | --- | --- | --- | --- | --- | --- | --- | --- | --- | --- | --- | --- | --- | --- | --- | --- | --- | --- | --- | --- | --- | --- | --- | --- | --- | --- | --- | --- | --- | --- | --- | --- | --- | --- | --- | --- | --- | --- | --- | --- | --- | --- | --- | --- | --- | --- | --- | --- | --- | --- | --- | --- | --- | --- | --- | --- | --- | --- | --- | --- | --- | --- | --- | --- | --- | --- | --- | --- | --- | --- | --- | --- | --- | --- | --- | --- | --- | --- | --- | --- | --- | --- | --- | --- | --- | --- | --- | --- | --- | --- | --- | --- | --- | --- | --- | --- | --- | --- | --- | --- | --- | --- | --- | --- | --- | --- | --- | --- | --- | --- | --- | --- | --- | --- | --- | --- | --- | --- | --- | --- | --- | --- | --- | --- | --- | --- | --- | --- | --- | --- | --- | --- | --- | --- | --- | --- | --- | --- | --- | --- | --- | --- | --- | --- | --- | --- | --- | --- | --- | --- | --- | --- | --- | --- | --- | --- | --- | --- | --- | --- | --- | --- | --- | --- | --- | --- | --- | --- | --- | --- | --- | --- | --- | --- | --- | --- | --- | --- | --- | --- | --- | --- | --- | --- | --- | --- | --- | --- | --- | --- | --- | --- | --- | --- | --- | --- | --- | --- | --- | --- | --- | --- | --- | --- | --- | --- | --- | --- | --- | --- | --- | --- | --- | --- | --- | --- | --- | --- | --- | --- | --- | --- | --- | --- | --- | --- | --- | --- | --- | --- | --- | --- | --- | --- | --- | --- | --- | --- | --- | --- | --- | --- | --- | --- | --- | --- | --- | --- | --- | --- | --- | --- | --- | --- | --- | --- | --- | --- | --- | --- | --- | --- | --- | --- | --- | --- | --- | --- | --- | --- | --- | --- | --- | --- | --- | --- | --- | --- | --- | --- | --- | --- | --- | --- | --- | --- | --- | --- | --- | --- | --- | --- | --- | --- | --- | --- | --- | --- | --- | --- | --- | --- | --- | --- | --- | --- | --- | --- | --- | --- | --- | --- | --- | --- | --- | --- | --- | --- | --- | --- | --- | --- | --- | --- | --- | --- | --- | --- | --- | --- | --- | --- | --- | --- | --- | --- | --- | --- | --- | --- | --- | --- | --- | --- | --- | --- | --- | --- | --- | --- | --- | --- | --- | --- | --- | --- | --- | --- | --- | --- | --- | --- | --- | --- | --- | --- | --- | --- | --- | --- | --- | --- | --- | --- | --- | --- | --- | --- | --- | --- | --- | --- | --- | --- | --- | --- | --- | --- | --- | --- | --- | --- | --- | --- | --- | --- | --- | --- | --- | --- | --- | --- | --- | --- | --- | --- | --- | --- | --- | --- | --- | --- | --- | --- | --- | --- | --- | --- | --- | --- | --- | --- | --- | --- | --- | --- | --- | --- | --- | --- | --- | --- | --- | --- | --- | --- | --- | --- | --- | --- | --- | --- | --- | --- | --- | --- | --- | --- | --- | --- | --- | --- | --- | --- | --- | --- | --- | --- | --- | --- | --- | --- | --- | --- | --- | --- | --- | --- | --- | --- | --- | --- | --- | --- | --- | --- | --- | --- | --- | --- | --- | --- | --- | --- | --- | --- | --- | --- | --- | --- | --- | --- | --- | --- | --- | --- | --- | --- | --- | --- | --- | --- | --- | --- | --- | --- | --- | --- | --- | --- | --- | --- | --- | --- | --- | --- | --- | --- | --- | --- | --- | --- | --- | --- | --- | --- | --- | --- | --- | --- | --- | --- | --- | --- | --- | --- | --- | --- | --- | --- | --- | --- | --- | --- | --- | --- | --- | --- | --- | --- | --- | --- | --- | --- | --- | --- | --- | --- | --- | --- | --- | --- | --- | --- | --- | --- | --- | --- | --- | --- | --- | --- | --- | --- | --- | --- | --- | --- | --- | --- | --- | --- | --- | --- | --- | --- | --- | --- | --- | --- | --- | --- | --- | --- | --- | --- | --- | --- | --- | --- | --- | --- | --- | --- | --- | --- | --- | --- | --- | --- | --- | --- | --- | --- | --- | --- | --- | --- | --- | --- | --- | --- | --- | --- | --- | --- | --- | --- | --- | --- | --- | --- | --- | --- | --- | --- | --- | --- | --- | --- | --- | --- | --- | --- | --- | --- | --- | --- | --- | --- | --- | --- | --- | --- | --- | --- | --- | --- | --- | --- | --- | --- | --- | --- | --- | --- | --- | --- | --- | --- | --- | --- | --- | --- | --- | --- | --- | --- | --- | --- | --- | --- | --- | --- | --- | --- | --- | --- | --- | --- | --- | --- | --- | --- | --- | --- | --- | --- | --- | --- | --- | --- | --- | --- | --- | --- | --- | --- | --- | --- | --- | --- | --- | --- | --- | --- | --- | --- | --- | --- | --- | --- | --- | --- | --- | --- | --- | --- | --- | --- | --- | --- | --- | --- | --- | --- | --- | --- | --- | --- | --- | --- | --- | --- | --- | --- | --- | --- | --- | --- | --- | --- | --- | --- | --- | --- | --- | --- | --- | --- | --- | --- | --- | --- | --- | --- | --- | --- | --- | --- | --- | --- | --- | --- | --- | --- | --- | --- | --- | --- | --- | --- | --- | --- | --- | --- | --- | --- | --- | --- | --- | --- | --- | --- | --- | --- | --- | --- | --- | --- | --- | --- | --- | --- | --- | --- | --- | --- | --- | --- | --- | --- | --- | --- | --- | --- | --- | --- | --- | --- | --- | --- | --- | --- | --- | --- | --- | --- | --- | --- | --- | --- | --- | --- | --- | --- | --- | --- | --- | --- | --- | --- | --- | --- | --- | --- | --- | --- | --- | --- | --- | --- | --- | --- | --- | --- | --- | --- | --- | --- | --- | --- | --- | --- | --- | --- | --- | --- | --- | --- | --- | --- | --- | --- | --- | --- | --- | --- | --- | --- | --- | --- | --- | --- | --- | --- | --- | --- | --- | --- | --- | --- | --- | --- | --- | --- | --- | --- | --- | --- | --- | --- | --- | --- | --- | --- | --- | --- | --- | --- | --- | --- | --- | --- | --- | --- | --- | --- | --- | --- | --- | --- | --- | --- | --- | --- | --- | --- | --- | --- | --- | --- | --- | --- | --- | --- | --- | --- | --- | --- | --- | --- | --- | --- | --- | --- | --- | --- | --- | --- | --- | --- | --- | --- | --- | --- | --- | --- | --- | --- | --- | --- | --- | --- | --- | --- | --- | --- | --- | --- | --- | --- | --- | --- | --- | --- | --- | --- | --- | --- | --- | --- | --- | --- | --- | --- | --- | --- | --- | --- | --- | --- | --- | --- | --- | --- | --- | --- | --- | --- | --- | --- | --- | --- | --- | --- | --- | --- | --- | --- | --- | --- | --- | --- | --- | --- | --- | --- | --- | --- | --- | --- | --- | --- | --- | --- | --- | --- | --- | --- | --- | --- | --- | --- | --- | --- | --- | --- | --- | --- | --- | --- | --- | --- | --- | --- | --- | --- | --- | --- | --- | --- | --- | --- | --- | --- | --- | --- | --- | --- | --- | --- | --- | --- | --- | --- | --- | --- | --- | --- | --- | --- | --- | --- | --- | --- | --- | --- | --- | --- | --- | --- | --- | --- | --- | --- | --- | --- | --- | --- | --- | --- | --- | --- | --- | --- | --- | --- | --- | --- | --- | --- | --- | --- | --- | --- | --- | --- | --- | --- | --- | --- | --- | --- | --- | --- | --- | --- | --- | --- | --- | --- | --- | --- | --- | --- | --- | --- | --- | --- | --- | --- | --- | --- | --- | --- | --- | --- | --- | --- | --- | --- | --- | --- | --- | --- | --- | --- | --- | --- | --- | --- | --- | --- | --- | --- | --- | --- | --- | --- | --- | --- | --- | --- | --- | --- | --- | --- | --- | --- | --- | --- | --- | --- | --- | --- | --- | --- | --- | --- | --- | --- | --- | --- | --- | --- | --- | --- | --- | --- | --- | --- | --- | --- | --- | --- | --- | --- | --- | --- | --- | --- | --- | --- | --- | --- | --- | --- | --- | --- | --- | --- | --- | --- | --- | --- | --- | --- | --- | --- | --- | --- | --- | --- | --- | --- | --- | --- | --- | --- | --- | --- | --- | --- | --- | --- | --- | --- | --- | --- | --- | --- | --- | --- | --- | --- | --- | --- | --- | --- | --- | --- | --- | --- | --- | --- | --- | --- | --- | --- | --- | --- | --- | --- | --- | --- | --- | --- | --- | --- | --- | --- | --- | --- | --- | --- | --- | --- | --- | --- | --- | --- | --- | --- | --- | --- | --- | --- | --- | --- | --- | --- | --- | --- | --- | --- | --- | --- | --- | --- | --- | --- | --- | --- | --- | --- | --- | --- | --- | --- | --- | --- | --- | --- | --- | --- | --- | --- | --- | --- | --- | --- | --- | --- | --- | --- | --- | --- | --- | --- | --- | --- | --- | --- | --- | --- | --- | --- | --- | --- | --- | --- | --- | --- | --- | --- | --- | --- | --- | --- | --- | --- | --- | --- | --- | --- | --- | --- | --- | --- | --- | --- | --- | --- | --- | --- | --- | --- | --- | --- | --- | --- | --- | --- | --- | --- | --- | --- | --- | --- | --- | --- | --- | --- | --- | --- | --- | --- | --- | --- | --- | --- | --- | --- | --- | --- | --- | --- | --- | --- | --- | --- | --- | --- | --- | --- | --- | --- | --- | --- | --- | --- | --- | --- | --- | --- | --- | --- | --- | --- | --- | --- | --- | --- | --- | --- | --- | --- | --- | --- | --- | --- | --- | --- | --- | --- | --- | --- | --- | --- | --- | --- | --- | --- | --- | --- | --- | --- | --- | --- | --- | --- | --- | --- | --- | --- | --- | --- | --- | --- | --- | --- | --- | --- | --- | --- | --- | --- | --- | --- | --- | --- | --- | --- | --- | --- | --- | --- | --- | --- | --- | --- | --- | --- | --- | --- | --- | --- | --- | --- | --- | --- | --- | --- | --- | --- | --- | --- | --- | --- | --- | --- | --- | --- | --- | --- | --- | --- | --- | --- | --- | --- | --- | --- | --- | --- | --- | --- | --- | --- | --- | --- | --- | --- | --- | --- | --- | --- | --- | --- | --- | --- | --- | --- | --- | --- | --- | --- | --- | --- | --- | --- | --- | --- | --- | --- | --- | --- | --- | --- | --- | --- | --- | --- | --- | --- | --- | --- | --- | --- | --- | --- | --- | --- | --- | --- | --- | --- | --- | --- | --- | --- | --- | --- | --- | --- | --- | --- | --- | --- | --- | --- | --- | --- | --- | --- | --- | --- | --- | --- | --- | --- | --- | --- | --- | --- | --- | --- | --- | --- | --- | --- | --- | --- | --- | --- | --- | --- | --- | --- | --- | --- | --- | --- | --- | --- | --- | --- | --- | --- | --- | --- | --- | --- | --- | --- | --- | --- | --- | --- | --- | --- | --- | --- | --- | --- | --- | --- | --- | --- | --- | --- | --- | --- | --- | --- | --- | --- | --- | --- | --- | --- | --- | --- | --- | --- | --- | --- | --- | --- | --- | --- | --- | --- | --- | --- | --- | --- | --- | --- | --- | --- | --- | --- | --- | --- | --- | --- | --- | --- | --- | --- | --- | --- | --- | --- | --- | --- | --- | --- | --- | --- | --- | --- | --- | --- | --- | --- | --- | --- | --- | --- | --- | --- | --- | --- | --- | --- | --- | --- | --- | --- | --- | --- | --- | --- | --- | --- | --- | --- | --- | --- | --- | --- | --- | --- | --- | --- | --- | --- | --- | --- | --- | --- | --- | --- | --- | --- | --- | --- | --- | --- | --- | --- | --- | --- | --- | --- | --- | --- | --- | --- | --- | --- | --- | --- | --- | --- | --- | --- | --- | --- | --- | --- | --- | --- | --- | --- | --- | --- | --- | --- | --- | --- | --- | --- | --- | --- | --- | --- | --- | --- | --- | --- | --- | --- | --- | --- | --- | --- | --- | --- | --- | --- | --- | --- | --- | --- | --- | --- | --- | --- | --- | --- | --- | --- | --- | --- | --- | --- | --- | --- | --- | --- | --- | --- | --- | --- | --- | --- | --- | --- | --- | --- | --- | --- | --- | --- | --- | --- | --- | --- | --- | --- | --- | --- | --- | --- | --- | --- | --- | --- | --- | --- | --- | --- | --- | --- | --- | --- | --- | --- | --- | --- | --- | --- | --- | --- | --- | --- | --- | --- | --- | --- | --- | --- | --- | --- | --- | --- | --- | --- | --- | --- | --- | --- | --- | --- | --- | --- | --- | --- | --- | --- | --- | --- | --- | --- | --- | --- | --- | --- | --- | --- | --- | --- | --- | --- | --- | --- | --- | --- | --- | --- | --- | --- | --- | --- | --- | --- | --- | --- | --- | --- | --- | --- | --- | --- | --- | --- | --- | --- | --- | --- | --- | --- | --- | --- | --- | --- | --- | --- | --- | --- | --- | --- | --- | --- | --- | --- | --- | --- | --- | --- | --- | --- | --- | --- | --- | --- | --- | --- | --- | --- | --- | --- | --- | --- | --- | --- | --- | --- | --- | --- | --- | --- | --- | --- | --- | --- | --- | --- | --- | --- | --- | --- | --- | --- | --- | --- | --- | --- | --- | --- | --- | --- | --- | --- | --- | --- | --- | --- | --- | --- | --- | --- | --- | --- | --- | --- | --- | --- | --- | --- | --- | --- | --- | --- | --- | --- | --- | --- | --- | --- | --- | --- | --- | --- | --- | --- | --- | --- | --- | --- | --- | --- | --- | --- | --- | --- | --- | --- | --- | --- | --- | --- | --- | --- | --- | --- | --- | --- | --- | --- | --- | --- | --- | --- | --- | --- | --- | --- | --- | --- | --- | --- | --- | --- | --- | --- | --- | --- | --- | --- | --- | --- | --- | --- | --- | --- | --- | --- | --- | --- | --- | --- | --- | --- | --- | --- | --- | --- | --- | --- | --- | --- | --- | --- | --- | --- | --- | --- | --- | --- | --- | --- | --- | --- | --- | --- | --- | --- | --- | --- | --- | --- | --- | --- | --- | --- | --- | --- | --- | --- | --- | --- | --- | --- | --- | --- | --- | --- | --- | --- | --- | --- | --- | --- | --- | --- | --- | --- | --- | --- | --- | --- | --- | --- | --- | --- | --- | --- | --- | --- | --- | --- | --- | --- | --- | --- | --- | --- | --- | --- | --- | --- | --- | --- | --- | --- | --- | --- | --- | --- | --- | --- | --- | --- | --- | --- | --- | --- | --- | --- | --- | --- | --- | --- | --- | --- | --- | --- | --- | --- | --- | --- | --- | --- | --- | --- | --- | --- | --- | --- | --- | --- | --- | --- | --- | --- | --- | --- | --- | --- | --- | --- | --- | --- | --- | --- | --- | --- | --- | --- | --- | --- | --- | --- | --- | --- | --- | --- | --- | --- | --- | --- | --- | --- | --- | --- | --- | --- | --- | --- | --- | --- | --- | --- | --- | --- | --- | --- | --- | --- | --- | --- | --- | --- | --- | --- | --- | --- | --- | --- | --- | --- | --- | --- | --- | --- | --- | --- | --- | --- | --- | --- | --- | --- | --- | --- | --- | --- | --- | --- | --- | --- | --- | --- | --- | --- | --- | --- | --- | --- | --- | --- | --- | --- | --- | --- | --- | --- | --- | --- | --- | --- | --- | --- | --- | --- | --- | --- | --- | --- | --- | --- | --- | --- | --- | --- | --- | --- | --- | --- | --- | --- | --- | --- | --- | --- | --- | --- | --- | --- | --- | --- | --- | --- | --- | --- | --- | --- | --- | --- | --- | --- | --- | --- | --- | --- | --- | --- | --- | --- | --- | --- | --- | --- | --- | --- | --- | --- | --- | --- | --- | --- | --- | --- | --- | --- | --- | --- | --- | --- | --- | --- | --- | --- | --- | --- | --- | --- | --- | --- | --- | --- | --- | --- | --- | --- | --- | --- | --- | --- | --- | --- | --- | --- | --- | --- | --- | --- | --- | --- | --- | --- | --- | --- | --- | --- | --- | --- | --- | --- | --- | --- | --- | --- | --- | --- | --- | --- | --- | --- | --- | --- | --- | --- | --- | --- | --- | --- | --- | --- | --- | --- | --- | --- | --- | --- | --- | --- | --- | --- | --- | --- | --- | --- | --- | --- | --- | --- | --- | --- | --- | --- | --- | --- | --- | --- | --- | --- | --- | --- | --- | --- | --- | --- | --- | --- | --- | --- | --- | --- | --- | --- | --- | --- | --- | --- | --- | --- | --- | --- | --- | --- | --- | --- | --- | --- | --- | --- | --- | --- | --- | --- | --- | --- | --- | --- | --- | --- | --- | --- | --- | --- | --- | --- | --- | --- | --- | --- | --- | --- | --- | --- | --- | --- | --- | --- | --- | --- | --- | --- | --- | --- | --- | --- | --- | --- | --- | --- | --- | --- | --- | --- | --- | --- | --- | --- | --- | --- | --- | --- | --- | --- | --- | --- | --- | --- | --- | --- | --- | --- | --- | --- | --- | --- | --- | --- | --- | --- | --- | --- | --- | --- | --- | --- | --- | --- | --- | --- | --- | --- | --- | --- | --- | --- | --- | --- | --- | --- | --- | --- | --- | --- | --- | --- | --- | --- | --- | --- | --- | --- | --- | --- | --- | --- | --- | --- | --- | --- | --- | --- | --- | --- | --- | --- | --- | --- | --- | --- | --- | --- | --- | --- | --- | --- | --- | --- | --- | --- | --- | --- | --- | --- | --- | --- | --- | --- | --- | --- | --- | --- | --- | --- | --- | --- | --- | --- | --- | --- | --- | --- | --- | --- | --- | --- | --- | --- | --- | --- | --- | --- | --- | --- | --- | --- | --- | --- | --- | --- | --- | --- | --- | --- | --- | --- | --- | --- | --- | --- | --- | --- | --- | --- | --- | --- | --- | --- | --- | --- | --- | --- | --- | --- | --- | --- | --- | --- | --- | --- | --- | --- | --- | --- | --- | --- | --- | --- | --- | --- | --- | --- | --- | --- | --- | --- | --- | --- | --- | --- | --- | --- | --- | --- | --- | --- | --- | --- | --- | --- | --- | --- | --- | --- | --- | --- | --- | --- | --- | --- | --- | --- | --- | --- | --- | --- | --- | --- | --- | --- | --- | --- | --- | --- | --- | --- | --- | --- | --- | --- | --- | --- | --- | --- | --- | --- | --- | --- | --- | --- | --- | --- | --- | --- | --- | --- | --- | --- | --- | --- | --- | --- | --- | --- | --- | --- | --- | --- | --- | --- | --- | --- | --- | --- | --- | --- | --- | --- | --- | --- | --- | --- | --- | --- | --- | --- | --- | --- | --- | --- | --- | --- | --- | --- | --- | --- | --- | --- | --- | --- | --- | --- | --- | --- | --- | --- | --- | --- | --- | --- | --- | --- | --- | --- | --- | --- | --- | --- | --- | --- | --- | --- | --- | --- | --- | --- | --- | --- | --- | --- | --- | --- | --- | --- | --- | --- | --- | --- | --- | --- | --- | --- | --- | --- | --- | --- | --- | --- | --- | --- | --- | --- | --- | --- | --- | --- | --- | --- | --- | --- | --- | --- | --- | --- | --- | --- | --- | --- | --- | --- | --- | --- | --- | --- | --- | --- | --- | --- | --- | --- | --- | --- | --- | --- | --- | --- | --- | --- | --- | --- | --- | --- | --- | --- | --- | --- | --- | --- | --- | --- | --- | --- | --- | --- | --- | --- | --- | --- | --- | --- | --- | --- | --- | --- | --- | --- | --- | --- | --- | --- | --- | --- | --- | --- | --- | --- | --- | --- | --- | --- | --- | --- | --- | --- | --- | --- | --- | --- | --- | --- | --- | --- | --- | --- | --- | --- | --- | --- | --- | --- | --- | --- | --- | --- | --- | --- | --- | --- | --- | --- | --- | --- | --- | --- | --- | --- | --- | --- | --- | --- | --- | --- | --- | --- | --- | --- | --- | --- | --- | --- | --- | --- | --- | --- | --- | --- | --- | --- | --- | --- | --- | --- | --- | --- | --- | --- | --- | --- | --- | --- | --- | --- | --- | --- | --- | --- | --- | --- | --- | --- | --- | --- | --- | --- | --- | --- | --- | --- | --- | --- | --- | --- | --- | --- | --- | --- | --- | --- | --- | --- | --- | --- | --- | --- | --- | --- | --- |
|  |  |  |  |  |  |  |  |  |  |  |  |  |  |  |  |
